# Associations of forest vs. urban environmental exposure with well-being and nasal microbiome composition: An exploratory pilot study

**DOI:** 10.1016/j.envres.2025.123582

**Published:** 2025-12-22

**Authors:** D. Connor Lashus, Andres Gomez, Thomas Hummel, Lucia F. Jacobs, Asifa Majid, Ravikiran M. Raju, Caroline J. Smith, Jonathan Williams, Gregory N. Bratman

**Affiliations:** a School of Environmental and Forest Sciences, University of Washington, Seattle, WA, 98195, USA; b Department of Animal Science, University of Minnesota, St. Paul, MN, 55108, USA; c Interdisciplinary Center Smell & Taste, Department of Otorhinolaryngology, Faculty of Medicine Carl Gustav Carus, Technische Universität Dresden, Dresden, Germany; d Department of Psychology, University of California, Berkeley, Berkeley, CA, 94720, USA; e Department of Experimental Psychology, University of Oxford, Oxford, UK; f Department of Brain and Cognitive Sciences, Picower Institute for Learning and Memory, Massachusetts Institute of Technology, Cambridge, MA, USA; g Division of Newborn Medicine, Harvard Medical School, Boston Children’s Hospital, Boston, MA, USA; h Department of Psychology and Neuroscience, Boston College, Chestnut Hill, MA, USA; i Max Planck Institute for Chemistry, 55128, Mainz, Germany; j Climate and Atmosphere Research Center, The Cyprus Institute, 1645, Nicosia, Cyprus; k Department of Psychology, University of Washington, Seattle, WA, 98195, USA; l Department of Environmental and Occupational Health Sciences, University of Washington, Seattle, WA, 98195, USA

**Keywords:** Nature exposure, Nasal microbiome, Affect, Rumination, Mental well-being, Aerobiome

## Abstract

The benefits of nature exposure for human well-being are well-recognized, yet much remains to be understood about the underlying causal mechanisms. This exploratory, hypothesis-generating pilot study used a natural experimental design with University of Washington students (Seattle, WA, USA; 2024) to investigate links between the nasal microbiome and well-being over an 8-week forest vs. urban environment exposure. After an academic year (September–May) during which all participants (*N* = 13) were full-time students in Seattle, one group relocated to remote forest sites in western Washington (*n* = 5; forest condition), while another group remained in urban Seattle (*n* = 8; urban condition). Self-reported affect, rumination, and mental well-being were assessed pre- and post-exposure using validated surveys, and nasal swabs were collected pre- and post-exposure for nasal microbiome profiling via 16S rRNA gene sequencing. Compared to the urban group, the forest group exhibited significantly greater increases in positive affect and decreases in negative affect and rumination. While no between-group differences in overall nasal bacterial community composition were detected pre-exposure, significant differences emerged post-exposure. Moreover, the forest group exhibited greater post-exposure taxonomic richness at a marginally statistically significant level and significant enrichment of taxa previously associated with well-being (e.g., *Bifidobacterium, Akkermansia*). These changes were not observed in the urban group. Increases in taxonomic richness and the relative abundance of these key taxa were significantly associated with affective improvements. These preliminary results suggest that nasal microbiome-mediated pathways linking nature exposure with well-being merit further investigation.

## 1. Introduction

The potential for natural environments to promote mental well-being and protect against mental illness has been acknowledged since antiquity and is reflected in Traditional Ecological Knowledge and other diverse cultural traditions. As early as the third millennium BCE, ancient Sumerians described gardens as places devoid of human illness (Bowe, 2017; van den Berg et al., 2018), and medieval European hospitals often integrated natural spaces believed to encourage mental and physical restoration (Marcus and Barnes, 1999). Building on these understandings, modern Western scientific approaches have provided empirical evidence for a causal link between exposure to natural environments and improvements in working memory and mood (Berman et al., 2012), as well as other affective benefits, such as decreased anxiety and rumination (Bratman et al., 2015, 2019; Hartig et al., 2014; Marselle et al., 2021; White et al., 2021). Proposed explanations for these effects include stress reduction (Ulrich et al., 1991), attention restoration (Kaplan, 1995), and modulation of immune and inflammatory activity (Andersen et al., 2021; Kuo, 2015; Li, 2010), though the biological pathways remain under active investigation (Elliott et al., 2023; Garrett et al., 2023). Given projections that 68% of the global population will reside in cities by 2050, developing a clear mechanistic understanding of the well-being benefits of nature exposure should be an urgent public health priority (United Nations, 2019).

Despite substantial progress in research on nature exposure and well-being in recent years, the potential mediating role of the human microbiome remains underexplored in these studies, with some notable exceptions (Lou et al., 2023; Prescott and Logan, 2017; Sobko et al., 2020; Varadarajan et al., 2025). This represents a critical gap given the environment’s strong influence on microbiome composition and mounting evidence linking microbial communities to psychological processes and behavior in other contexts (Rothschild et al., 2018; Sarkar et al., 2018; Sudo et al., 2004).

Research demonstrates that gut microbial communities can influence neuropsychological outcomes through immune, neural, and endocrine pathways—an insight that has shaped the broader concept of “psychobiotics,” or microbes capable of supporting host mental health (Cheng et al., 2019; Dinan et al., 2013; Hashimoto, 2023; Mosquera et al., 2024; Sarkar et al., 2016; R. Sharma et al., 2021). Key mechanisms include microbial production of short-chain fatty acids (SCFAs), which promote epithelial barrier integrity, regulate local and systemic immune response, and modulate expression of neuroplasticity-related genes, as well as microbial modulation of available neuroactive compounds (Dalile et al., 2019; Wall et al., 2014; Zhou et al., 2023). Genera frequently implicated in these mechanisms—such as *Bifidobacterium, Lactobacillus,* and *Akkermansia*—are often regarded as markers of a healthy gut microbiome (Ding et al., 2021; Sarkar et al., 2016).

In contrast to the gut microbiome, however, the nasal microbiome remains underexplored in relation to psychobiotic mechanisms. The olfactory pathway represents a sensory interface deeply tied to mental well-being, where volatile compounds from the environment influence the CNS through subjective experiences and other psychoneuroimmunological processes that may bypass conscious detection (Bratman et al., 2024; Dikeçligil and Gottfried, 2024; Kumpitsch et al., 2019; Taufer et al., 2025; Thangaleela et al., 2022). Notably, the olfactory pathway can also provide a route for molecules to bypass the blood-brain barrier, enabling inhaled molecules and microbial metabolites from the olfactory epithelium to reach the brain (Fayet-Moore and Robinson, 2024).

Beyond direct effects of volatile compounds through the olfactory pathway, growing evidence indicates that nasal microbial communities are shaped by chemical and microbial exposures from the environment (Dalton et al., 2022; Kraemer et al., 2018, 2021; Zhang et al., 2023). Recent studies have begun examining relationships between natural environment exposure (e.g., residential surrounding greenspace) and nasal microbiome composition (Asri et al., 2023; Brame et al., 2024; Gisler et al., 2021; Pearson et al., 2019, 2020; Selway et al., 2020; Zhang et al., 2024), although results to date are mixed. The nasal microbiome has also been linked to olfactory function and respiratory health (Biswas et al., 2020; Cho et al., 2020; Hoggard et al., 2017; Konovalovas et al., 2024; Koskinen et al., 2018), but its relevance to mental well-being is a relatively new area of research (Hashimoto, 2023; Hoggard et al., 2018; Lazarini et al., 2022; Song et al., 2025; Vásquez-Pérez et al., 2024). Further, while several gut microbiome studies have integrated measures of nature exposure, microbiome composition, and mental well-being (Brame et al., 2021; Liddicoat et al., 2020; Sobko et al., 2020), similar investigations of the nasal microbiome are needed to define its potential mediating role of the well-being benefits of nature exposure.

To begin to address this gap, we conducted an exploratory pilot study examining associations of an 8-week forest vs. urban environment exposure with changes in (1) affect, rumination, and mental well-being, and (2) nasal microbiome composition. We also assessed associations between the two. In an additional exploratory analysis, we assessed the pre- and post-exposure compositional similarity of participants’ nasal microbiomes with the forest aerobiome, as emerging research suggests that airborne microbial exposures—particularly in natural biodiverse environments—may shape human respiratory microbiota and influence health outcomes (Fayet-Moore and Robinson, 2024).

We hypothesized that: (***H1***) forest vs. urban environment exposure would be associated with larger improvements in self-reported affect, rumination, and mental well-being measures, as well as with greater increases in nasal bacterial richness and SDI; (***H2***) overall nasal bacterial community composition would differ between groups post-exposure—but not pre-exposure—and bacteria often associated with forest environments would be associated with forest group nasal microbiome post-exposure samples; (***H3***) compositional overlap between the forest—but not urban—group’s microbiomes and the forest aerobiome would increase over the exposure period (***H4***) taxa associated with well-being benefits (e.g., immunoregulation, epithelial barrier maintenance, neuroprotection); would be enriched in the forest—but not urban—group post-exposure; and (***H5***) increases in nasal bacterial α-diversity and the relative abundance of some specific taxa linked to well-being benefits in other research would be associated with improvements in the well-being outcomes assessed in this pilot study.

## 2. Materials & methods

### 2.1. Study design and participants

The study followed a natural experimental design comparing two groups of healthy adult students from the University of Washington (Seattle, USA) who experienced contrasting environmental conditions over an 8-week summer academic term. All participants (*N* = 13) were full-time, on-campus students for the preceding academic year (September–May). Inclusion criteria included non-smoker, no current diagnosis of a psychiatric or neurological condition, no acute illness or infection in the month prior to enrollment, and no antibiotic use in the month prior to enrollment. The urban environment group (*n* = 8; 6 female, 2 male, mean age 22y) remained in Seattle for the 8-week period, working or attending classes on campus. The forest environment group (*n* = 5; 4 female, 1 male, mean age 21y) relocated from Seattle to remote western Washington forests, where they lived on-site and worked outdoors 30+ hours/week on forestry projects. Four forest group participants were based at the Olympic Natural Resources Center (ONRC) near Forks, WA, and one at the Center for Sustainable Forestry at Pack Forest (PF) near Eatonville, WA. Both forest locations are substantially different from Seattle’s urban setting. Each is remote from major urban centers and surrounded by temperate coniferous forest dominated by Douglas-fir, hemlock, and Sitka spruce, which is typical of Washington forest ecosystems west of the Cascade Mountains (Palmer et al., 2019). All study sites are below 250 m elevation. Average temperature/relative humidity over the study period was 68°F/64% in Seattle, 62°F/84% in Forks, and 60°F/71% in Eatonville (National Centers for Environmental Information, 2024; National Oceanic and Atmospheric Administration, 2024). Because this was a natural experimental design, participants self-selected into their summer environments rather than being randomly assigned.

Participants were assessed at two timepoints, corresponding to pre- and post-exposure measurements. The first assessment occurred at the end of the spring academic quarter on the University of Washington’s Seattle campus. The second assessment took place eight weeks later—for the urban group, in the same location, and for the forest group, at their respective forest sites prior to departure. At both timepoints, participants completed self-reported well-being surveys and provided nasal swabs for nasal microbiome sequencing. Each session lasted approximately 1 h. Participants received $50/session plus a $20 study completion bonus. All participants provided written informed consent prior to their enrollment. The study protocol was approved by the UW Institutional Review Board (STUDY00020149; June 26, 2024).

### 2.2. Affect, rumination, and mental well-being surveys

All survey data were collected and managed using Research Electronic Data Capture (REDCap) hosted at the University of Washington (Harris et al., 2009, 2019). REDCap is a secure, web-based platform designed to support data capture for research studies providing an intuitive interface for validated data capture; audit trails for tracking data manipulation and export procedures; automated export procedures for seamless data downloads to common statistical packages; and procedures for data integration and interoperability with external sources.

Positive and negative affect were assessed using the 20-item Positive and Negative Affect Schedule (PANAS) (Watson et al., 1988), which includes two 10-item subscales: a positive affect scale (e.g., “enthusiastic”, “inspired”) and a negative affect scale (e.g., “ashamed”, “nervous”). Participants rated the extent to which they experienced each feeling or emotion over the past two weeks on a 5-point Likert scale ranging from “Very slightly or not at all” to “Extremely.” Higher scores on each subscale indicated greater positive or negative affect, respectively. Both subscales were internally consistent (positive affect α = 0.89; negative affect α = 0.87).

Rumination was assessed using the 12-item rumination subscale of the Rumination-Reflection Questionnaire (RRQ) (Trapnell and Campbell, 1999), which assesses the tendency toward repetitive, self-critical thought (e.g., “I often find myself re-evaluating something I’ve done”). Participants rated each item on a 5-point Likert scale ranging from “Strongly disagree” to “Strongly agree.” Higher scores indicate greater ruminative tendency. The scale was internally consistent (α = 0.89).

Mental well-being was assessed using the 14-item Warwick-Edinburgh Mental Wellbeing Scale (WEMWBS) (Tennant et al., 2007), which captures positive domains of psychological well-being (e.g., “I’ve been feeling optimistic about the future”). Participants rated each item on a 5-point Likert scale ranging from “None of the time” to “All of the time,” reflecting their experience over the last two weeks. Higher scores indicate greater mental well-being. The scale was internally consistent (α = 0.92).

### 2.3. Nasal microbiome sampling

At each timepoint, gloved participants self-collected four nasal swab samples under supervision of the study team—two from the left anterior naris and two from the right—using sterile, individually wrapped foam-tipped swabs (Puritan^®^). Each swab was inserted approximately 1 cm into the nostril and rotated along the inner wall for 30 s. The participant then inserted the swab tip into a 2 mL Eppendorf Safe-Lock^®^ tube containing 1 mL of DNA/RNA Shield (Zymo Research). Following collection, samples were stored temporarily at −20 °C and transferred to −80 °C at University of Washington’s Microbial Interactions and Microbiome Center (mim_C) within 24 h of collection.

### 2.4. Aerobiome sampling

Airborne bacteria were collected using the Coriolis^®^ Compact air sampler (Bertin Technologies). For each collection, the instrument was positioned approximately 1 m above the ground, and airborne particles were collected and vortexed into a non-sterile, single-use collection cone for 20 min at an airflow rate of 50 L/min. Due to supply chain issues, sterile collection cones were not available for this pilot project. Cones were used as received from the manufacturer. After sampling, cones were immediately washed with 1 mL of DNA/RNA Shield (Zymo Research) and placed in temporary storage at −20 °C before transfer to −80 °C at mim_C within 24 h of collection. To monitor potential contamination, one DNA extraction control blank was processed alongside four forest air samples following identical extraction and sequencing procedures, and samples were screened for contaminants using the decontam package (Davis et al., 2018).

### 2.5. DNA extraction and sequencing

Microbial DNA was extracted from nasal swab and air samples using the QIAamp^®^ DNA Mini Kit (Qiagen) according to the manufacturer’s protocol for DNA purification from blood or body fluids (spin protocol). Final elution volume was reduced from 2 × 200 μL to 2 × 50 μL to increase DNA concentration. Amplicon libraries of the 16S rRNA gene V3-V4 region were prepared using primers S-D-Bact-0341-b-S-17 and S-D-Bact-0785-a-A-21(Klindworth et al., 2013) in accordance with Illumina’s recommendations for 16S Metagenomic Sequencing Library Preparation (Part# 15044223 Rev. B). Libraries were sequenced using Illumina NextSeq2000^®^ with 600 cycle X-LEAP chemistries. After demultiplexing, paired end sequences were processed using the QIIME2 pipeline (v. 2023.9.1) (Bolyen et al., 2019). Briefly, primers were trimmed using the cutadapt plugin (v. 4.5) (Martin, 2011). Denoising, quality filtering, and enumeration of amplicon sequence variants (ASVs) was performed using the DADA2 plugin, also within QIIME2 (Callahan et al., 2016). Taxonomic assignments were established using scikit-learn naïve bayes classifier trained on the SILVA SSU Ref NR99 138.1 dataset (Quast et al., 2013).

### 2.6. Statistical analyses

All statistical analyses were conducted in the R interface (R Core Team, 2024). Changes in affect, rumination, mental well-being, and α-diversity metrics were assessed using linear mixed effects models (LMM) implemented in the lme4 package (Bates et al., 2015), with statistical significance evaluated using the lmerTest package (Kuznetsova et al., 2017). Each model included fixed effects of group (forest vs. urban), time (pre- vs. post-exposure), and their interaction, with a random intercept for participant ID to account for repeat measures within individuals. Associations between changes in well-being and compositional microbiome metrics were assessed using linear regression models implemented in the rigr package (Chen et al., 2022). Basic regression diagnostics using Q-Q plots and Cook’s distance were performed to inspect residuals and identify potentially influential observations.

Within-sample microbiome diversity (α-diversity) metrics—ASV richness and Shannon diversity index (SDI)—were calculated using the phyloseq package (McMurdie and Holmes, 2013). Between-sample diversity (β-diversity) metrics—unweighted Jaccard and weighted Bray-Curtis dissimilarity—were calculated using the vegan package (Oksanen et al., 2025). Differences in community composition across groups and timepoints were evaluated using permutational multivariate analysis of variance (PERMANOVA) using the adonis2 function with 999 permutations. Permutational analysis of multivariate dispersion (PERMDISP) based on dissimilarity indices was used to test for homogeneity of group dispersion. β-diversity results were visualized using non-metric multidimensional scaling (NMDS) ordinations. Differentially abundant bacterial taxa across groups and timepoints were identified using the DESeq2 package (Love et al., 2014) with Wald tests and false discovery rate (FDR) correction (Benjamini and Hochberg, 1995). Indicator taxa analysis using the labdsv package (Roberts, 2023) was used as a complementary analysis to identify taxa associated with specific environmental conditions pre- and post-exposure. Box, bar, and ordination plots were produced using the ggplot2 package (Wickham, 2016), and Venn diagrams were created using the eulerr package (Larsson, 2024).

## 3. Results

### 3.1. Participant characteristics

The demographic details of participants can be found in Table 1. There were no significant differences in age, Welch’s *t*-test, *t*(10.12) = 1.35, *p* = .21, sex at birth, Fisher’s Exact Test *p* = 1, or race/ethnicity, Fisher’s Exact Test *p* = .27 across groups. There were also no baseline group differences in self-reported dietary pattern (i.e., omnivorous, vegetarian, vegan), Fisher’s Exact Test *p* = 1, or alcohol use frequency, Fisher’s Exact Test *p* = .42. Finally, there were no baseline group differences in self-reported hours per week of nature exposure, Welch’s *t*-test, *t*(6.16) = 0.98, *p* = .37 (forest mean = 19.60 h, urban mean = 13.13 h). However, post-exposure, the forest group reported significantly greater weekly nature exposure than the urban group, Welch’s *t*-test, *t*(5.64) = 5.40, *p* < .01 (forest mean = 67.60 h, urban mean 14.25 h) (Fig. S1). More information about the settings in which participants reported spending most of their nature exposure time is also available in the Supplementary Materials (Fig. S2).

### 3.2. H1: Forest exposure is associated with affective and rumination outcomes

Changes in affect, rumination, and mental well-being were assessed using LMM with fixed effects of group (forest vs. urban), time (pre-vs. post-exposure), and their interaction, and a random intercept for participant. Significant group × time interactions indicate differential change over time between groups. When interactions were significant, we conducted planned, pairwise *t*-tests to examine pre-to post-exposure change within each group. This approach allowed us to determine whether an observed interaction was driven by significant gains in the forest group, losses in the urban group, or a combination of both. Pre-exposure group differences were also assessed using Welch’s *t*-tests; none were significant (all *p*s > .11). Mean scores for all survey measures, as well as full model results, can be found in the Supplementary Materials (Tables S1 and S2).

**Fig. 1.**
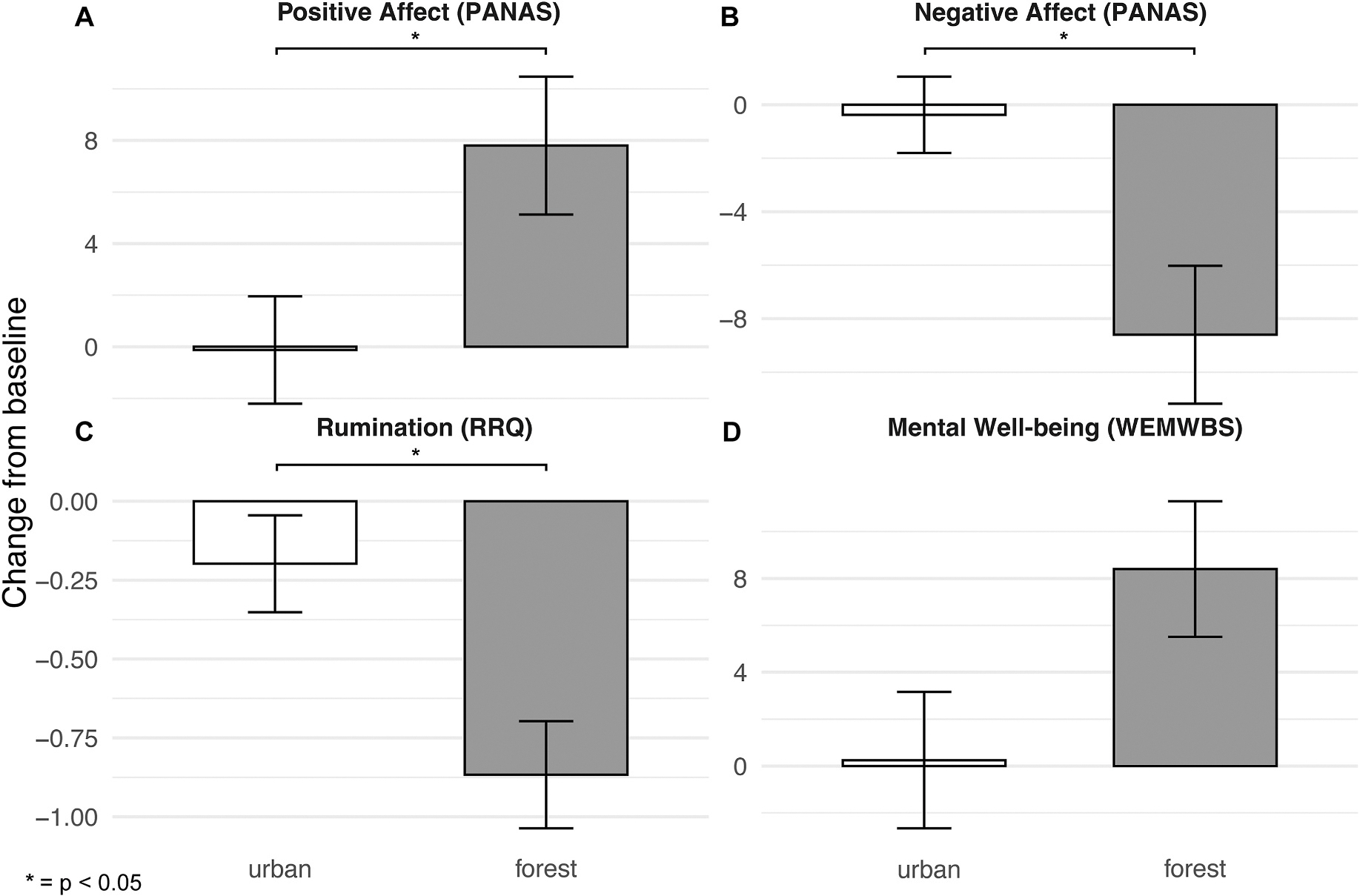
Affect, rumination, and mental well-being survey score changes from baseline by group. Each panel shows pre- to post-exposure change in survey score for the urban (*n* = 8) and forest groups (*n* = 5) on one of four psychological measures: (A) positive affect, (B) negative affect, (C) rumination, and (D) mental well-being. Positive values indicate an increase in score over the study period, and negative values indicate a decrease. Error bars represent standard error (SE) values.

The forest group exhibited significantly greater increases in positive affect and decreases in negative affect and rumination over the 8-week period compared to the urban group (Fig. 1). The greater increase in WEMWBS score for the forest (vs. urban) group was trend-level and not statistically significant. We report statistics for each of these findings below.

#### 3.2.1. Positive affect

The LMM of positive affect (PANAS) revealed a significant group × time interaction, *β* = 7.93, 95% CI: 1.31–14.54, SE = 3.38, *p* = .04, with no main effect of time or group. Follow-up pairwise *t*-tests revealed no change over time for the urban group, *t*(7) = −0.06, *p* = .95, but a significant increase for the forest group, *t*(4) = 2.92, *p* = .04. Together, these findings demonstrate that the interaction was driven primarily by increases in positive affect for the forest group.

#### 3.2.2. Negative affect

The LMM of negative affect (PANAS) revealed a significant main effect of group, *β* = 7.15, 95% CI: 1.38–12.92, SE = 2.94, *p* = .03, and a significant group × time interaction, *β* = −8.23, 95% CI: −13.52 – −2.93, SE = 2.70, *p* = .01, but no significant effect of time. The main effect of group indicates that, across timepoints, mean negative affect differed between groups. However, as stated above, Welch’s *t*-tests revealed no significant differences pre-exposure, *t*(5.37) = −1.73, *p* = .14. Follow-up pairwise *t*-tests revealed no change over time for the urban group, *t*(7) = −0.26, *p* = .80, but a significant decrease for the forest group, *t*(4) = −3.33, *p* = .03. Together, these findings demonstrate that the interaction was driven primarily by decreases in negative affect for the forest group.

#### 3.2.3. Rumination

The LMM of rumination (RRQ) revealed a significant group × time interaction, *β* = −0.67, 95% CI: −1.13 to – −0.20, SE = 0.24, *p* = .02, with no main effect of time or group. Follow-up pairwise *t*-tests revealed no change over time for the urban group, *t*(7) = −1.29, *p* = .24, but a significant decrease for the forest group, *t*(4) = −5.01, *p* = .01. Together, these findings demonstrate that the interaction was driven primarily by decreases in rumination for the forest group.

#### 3.2.4. WEMWBS

The LMM of mental well-being (WEMWBS) revealed a trend-level group × time interaction, *β* = 8.15, 95% CI: −0.38 – 16.68, SE = 4.35, *p* = .09, with no main effect of either group or time. Follow-up pairwise *t*-tests revealed no change over time for the urban group, *t*(7) = 0.09, *p* = .93, but a significant increase for the forest group, *t*(4) = 2.91, *p* = .04. While the interaction did not reach statistical significance, and thus we cannot reject the null hypothesis that change in mean score from pre- to post-exposure was equivalent for the urban and forest groups, these results suggest a differential pattern wherein the forest group showed increased mental well-being over time while the urban group did not.

### 3.3. H1 and 2: Forest exposure is associated with nasal bacterial community changes

We next examined compositional changes in participants’ nasal bacterial communities over the exposure period. In total, 26 nasal swab samples were included in the analyses – one pre- and one post-exposure sample per participant. Sequencing of the V3-V4 region of the 16S rRNA gene identified 1550 ASVs. The predominant phyla observed across all samples were *Proteobacteria* (23%), *Actinobacteria* (18%), *Firmicutes* (18%), and *Bacteroidota* (14%), which is typical of the human nasal microbiome (Koskinen et al., 2018). Twenty-eight variants identified as mitochondrial or chloroplast DNA were removed prior to further analysis. Rarefaction curves (see Fig. S3 in the Supplementary Materials) indicate sufficient sequencing depth to capture the majority of bacterial diversity present in the samples.

We first assessed change in bacterial α-diversity (ASV richness and SDI) over time and between groups. LMMs tested for group × time interactions indicative of differential change in α-diversity over the exposure period. Planned Wilcoxon tests compared groups at each timepoint. Group means and full model results are available in the Supplementary Materials (Tables S3 and S4). Results of a complementary analysis of covariance (ANCOVA) adjusting for baseline α-diversity values are available in the Supplementary Materials (Table S5).

**Fig. 2.**
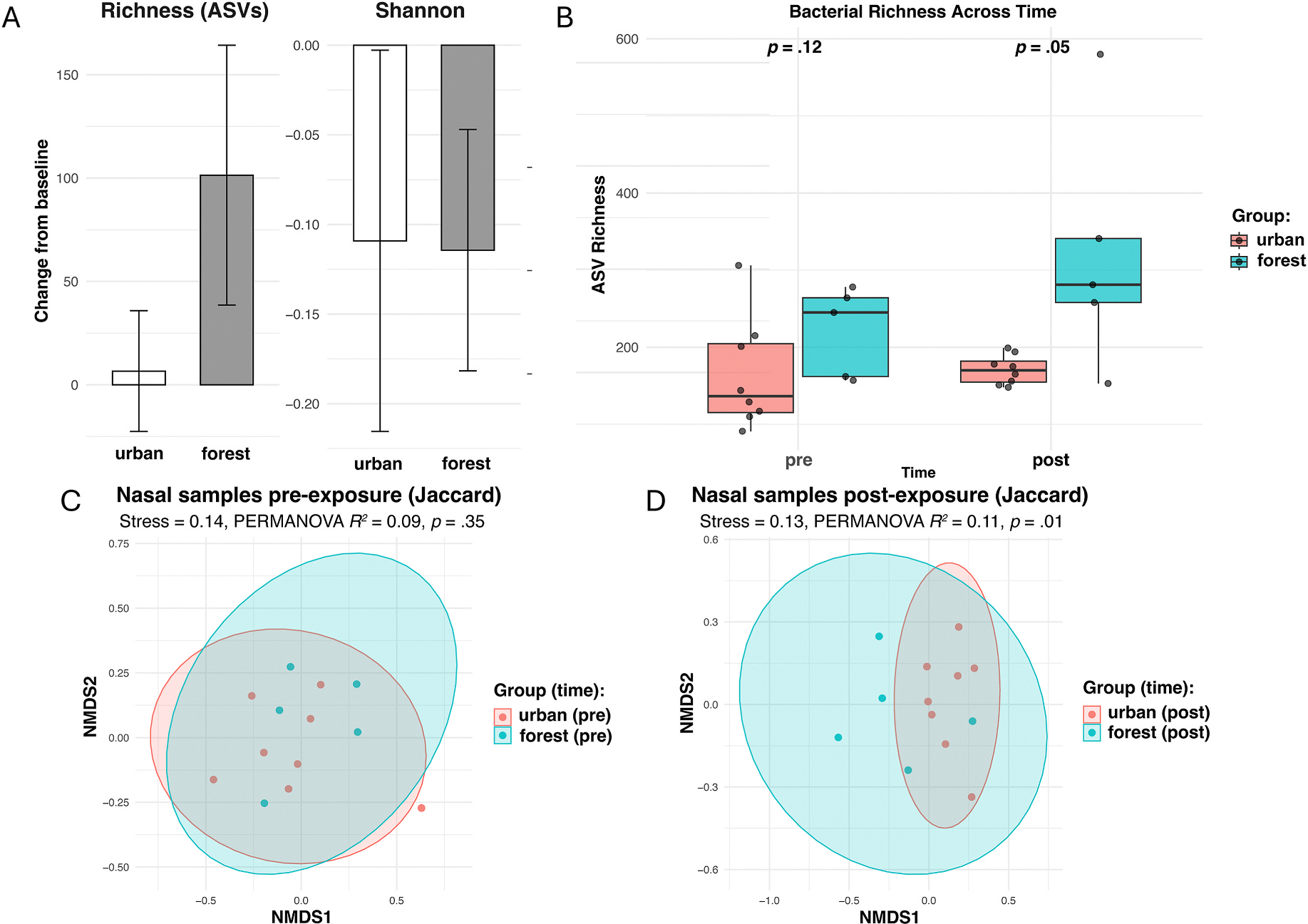
Pre- to post-exposure differences in nasal bacterial community diversity. (A) Change from baseline in ASV richness and SDI for urban (*n* = 8) and forest groups (*n* = 5) (error bars represent standard error). (B) ASV richness for urban and forest groups across timepoints (*p*-values from Wilcoxon rank-sum test). (C–D) Non-metric multidimensional scaling (NMDS) ordination of nasal bacterial communities based on unweighted Jaccard distances pre- (C) and post-exposure (D), with ellipses representing 95% confidence intervals around group centroids. PERMANOVA results indicate no significant difference in community composition pre-exposure, followed by a significant difference post-exposure, and the forest group exhibits higher post-exposure richness.

The LMM of ASV richness revealed no significant main effects of group or time and no significant group × time interaction, *β* = 94.78, 95% CI: −25.20 – 214.75, SE = 61.21, *p* = .15, (Fig. 2A). Follow-up Wilcoxon rank-sum tests (Fig. 2B) revealed no difference between groups pre-exposure, *W* = 9, *p* = .12, but a marginally significant difference post-exposure, *W* = 6, *p* = .05. The forest group exhibited higher post-exposure mean richness (322.60 ASVs) than the urban group (170.75 ASVs), though there was substantial variability across individuals. Within-group comparisons using Wilcoxon signed-rank tests revealed no significant change in mean richness over time in either the forest, *V* = 12, *p* = .28, or urban group, *V* = 21, *p* = .73. Together, these results suggest a potential trend toward higher bacterial ASV richness in the forest vs. urban group post-exposure, though this interpretation should be made cautiously given the nonsignificant interaction. We cannot reject the null hypothesis that change in ASV richness over time was equivalent across groups.

The LMM of SDI, which reflects both richness and evenness of taxa in a sample, revealed no significant main effects of group or time and no significant group × time interaction, all *p*s ≥ .25 (Fig. 2B). Follow-up Wilcoxon rank-sum tests revealed no difference between groups pre- or post-exposure, all *p*s ≥ .51, and Wilcoxon signed-rank tests revealed no change over time in either group, all *p*s ≥ .18. These results suggest that the forest group’s higher post-exposure richness may reflect the addition of low-abundance taxa, increasing the total number of detected taxa but not markedly changing the overall distribution of taxa within the nasal microbiome.

We further assessed differences in nasal bacterial community composition across groups and time using unweighted (Jaccard) and weighted (Bray-Curtis) β-diversity metrics. Based on Jaccard dissimilarity, PERMANOVA results showed no significant difference between groups pre-exposure, *R*^*2*^ = .09, *p* = .35 (Fig. 2C), but a significant difference post-exposure, *R*^*2*^ = .11, *p* = .01. NMDS visualization indicated moderate separation between groups post-exposure with considerable overlap in 95% confidence ellipses (Fig. 2D), consistent with the small sample size. PERMDISP revealed no significant difference in multivariate dispersion between groups, *p* = .82, suggesting that the PERMANOVA result reflects genuine compositional differences rather than unequal within-group variability. Within-group unweighted comparisons of pre- and post-exposure samples were non-significant for both the forest, *R*^*2*^ = .12, *p* = .30, and urban group, *R*^*2*^ = .08, *p* = .10.

PERMANOVA based on Bray-Curtis dissimilarity revealed no significant differences in weighted β-diversity across groups or time (Fig. S4), suggesting that the observed post-exposure differences were primarily compositional rather than structural. This pattern aligns with the α-diversity results and reflects the distinction between unweighted and weighted β-diversity metrics. Whereas the unweighted Jaccard results indicate a post-exposure difference between groups in which taxa were detected, the nonsignificant Bray-Curtis results indicate that relative abundances of taxa were largely stable. Together, these findings suggest that post-exposure differences may reflect the addition of low-abundance taxa in the forest group, altering community composition without substantially changing the overall structure or evenness of the nasal microbiome.

To interrogate this further, we conducted taxon-level analyses of presence-absence data. A Fisher’s exact test identified 592 ASVs with significantly different presence across groups post-exposure, FDR-adjusted *p* < .05, indicating group-level compositional divergence. Indicator taxa analysis across four sample conditions—urban pre-exposure, urban post-exposure, forest pre-exposure, and forest post-exposure—identified 24 ASVs significantly associated with forest group post-exposure samples. Most were taxa typically associated with soil or plant environments, and all were present at very low relative abundance (<1% of the community, on average) (Table S6). These findings suggest that post-exposure differences in bacterial community composition between groups may have been influenced by the forest group’s acquisition of low-abundance, forest environment-derived taxa, though additional research is necessary to substantiate these results and this preliminary interpretation.

### 3.4. H2 and 4: Forest exposure is associated with shifts in the relative abundance of bacterial taxa

To investigate the magnitude of taxon-specific change over time, we then performed differential abundance analysis, identifying ASVs whose relative abundance changed significantly from pre-to post-exposure within each group. Separate analyses were conducted for the forest and urban groups, with FDR correction applied to account for multiple testing.

**Fig. 3.**
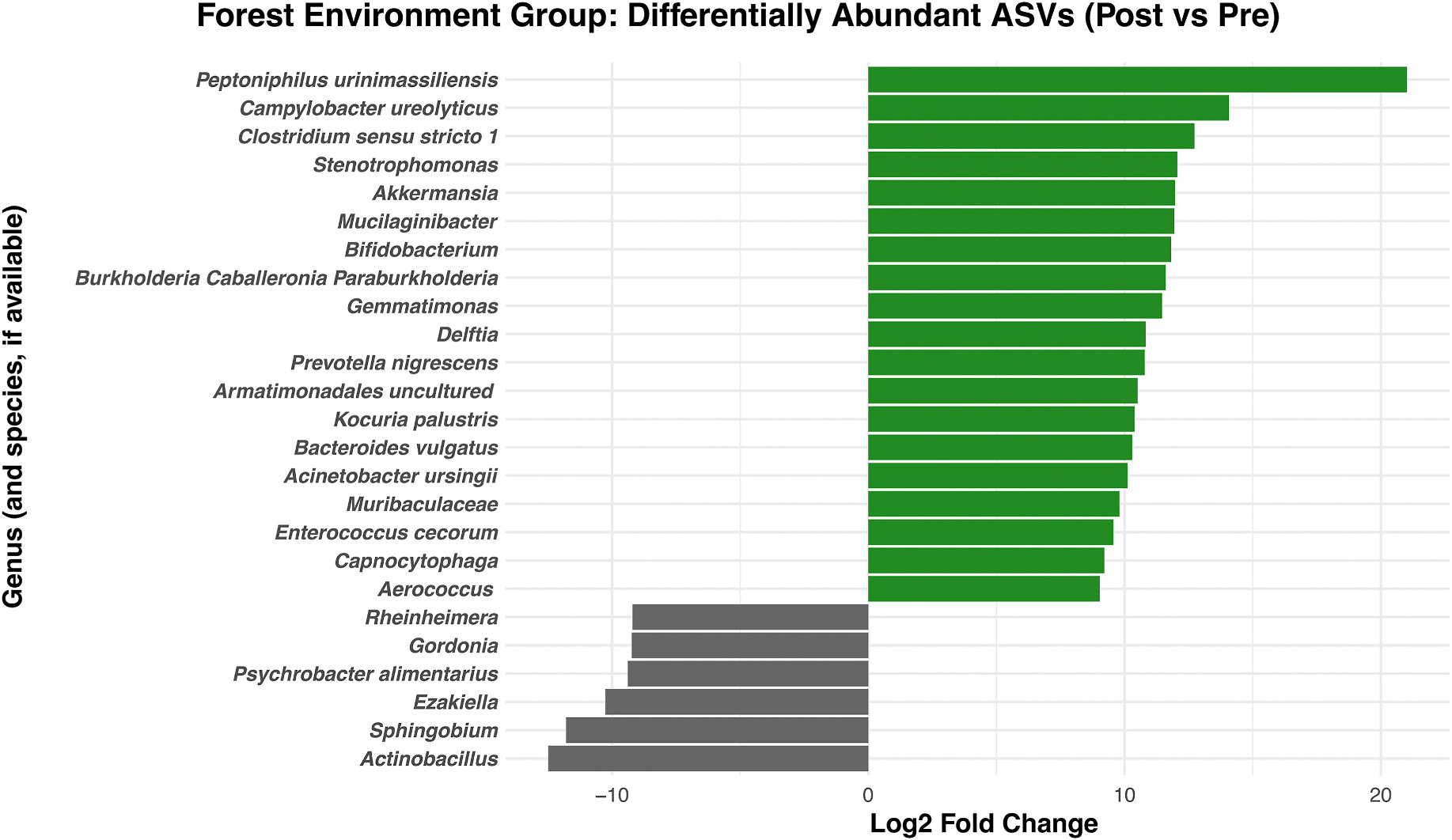
Differentially abundant bacterial taxa in the forest group from pre- to post-exposure. Log_2_ fold changes in ASV relative abundance over the study period for 19 enriched and 6 depleted taxa (FDR-adjusted *p* < .05). Positive values (green bars) indicate taxa that increased in relative abundance in the forest group’s nasal microbiomes from pre- to post-exposure, while negative values (gray bars) indicate taxa that decreased in relative abundance. Several enriched genera have been associated with well-being-promoting functions (e.g., *Akkermansia, Bifidobacterium*), while others represent environmental taxa not typically found in the nasal microbiota (e.g., *Mucilaginibacter, Gemmatimonas*).

In the forest group, 19 ASVs identifiable to at least the genus level were enriched and 6 ASVs were depleted over the exposure period, FDR-adjusted *p* < .05 (Fig. 3). *Peptoniphilus urinimassiliensis,* a minimally characterized species isolated from human urine (Brahimi et al., 2017), was the most differentially abundant. Notably, several enriched genera – *Bifidobacterium, Akkermansia, Muribaculaceae, and Clostridium sensu stricto 1* – are associated with beneficial immunoregulatory, epithelial barrier-protective, and neuromodulatory effects (Derrien et al., 2011; Ma et al., 2022; Tian et al., 2021; Zhu et al., 2024). The forest group was also enriched in genera common in soil, plant, or water environments, such as *Mucilaginibacter and Gemmatimonas*. These are not typically associated with the human nasal microbiota but have the potential to produce bioactive metabolites like terpenes and SCFAs (Figueiredo et al., 2022; Zheng et al., 2022). *Campylobacter ureolyticus*, a pathogen typically associated with zoonotic or foodborne gastroenteritis (Maki et al., 2023), was also enriched, highlighting the inherent complexity of environmental microbial exposures and the possibility that such exposures may involve taxa with varied ecological or clinical associations.

Notably, none of these same ASVs were enriched in the urban group (Fig. S5). The genus *Stenotrophomonas,* which is broadly distributed across a wide range of environments (Ryan et al., 2009), was the only taxon to exhibit increased abundance over time in both groups. The most significantly depleted ASV in the forest group over time was of the genus *Actinobacillus,* which includes several mammalian pathogens and has been linked to upper airway inflammation in humans (Bossé et al., 2022; Ma et al., 2024). The enrichment of taxa previously associated with immunomodulatory and other well-being-beneficial functions in the forest group suggests that forest vs. urban environmental context may influence the relative abundance of potentially health-promoting bacteria in the nasal passages.

### 3.5. H3: Forest group’s nasal microbiomes share more taxa with the forest aerobiome post-vs. pre-exposure

Because aerobiome samples were collected using non-sterile cones, these results should be interpreted with considerable caution and are reported as exploratory. Contaminant screening using a prevalence-based algorithm did not flag any ASVs as contaminants, which likely reflects the low biomass of aerobiome samples, limited statistical power provided by a single control, or compositional similarity between samples and control rather than an absence of contamination. Given these constraints, our analyses emphasized relative compositional patterns between nasal and forest air smaples rather than interpretation of absolute aerobiome taxonomic profiles.

**Fig. 4.**
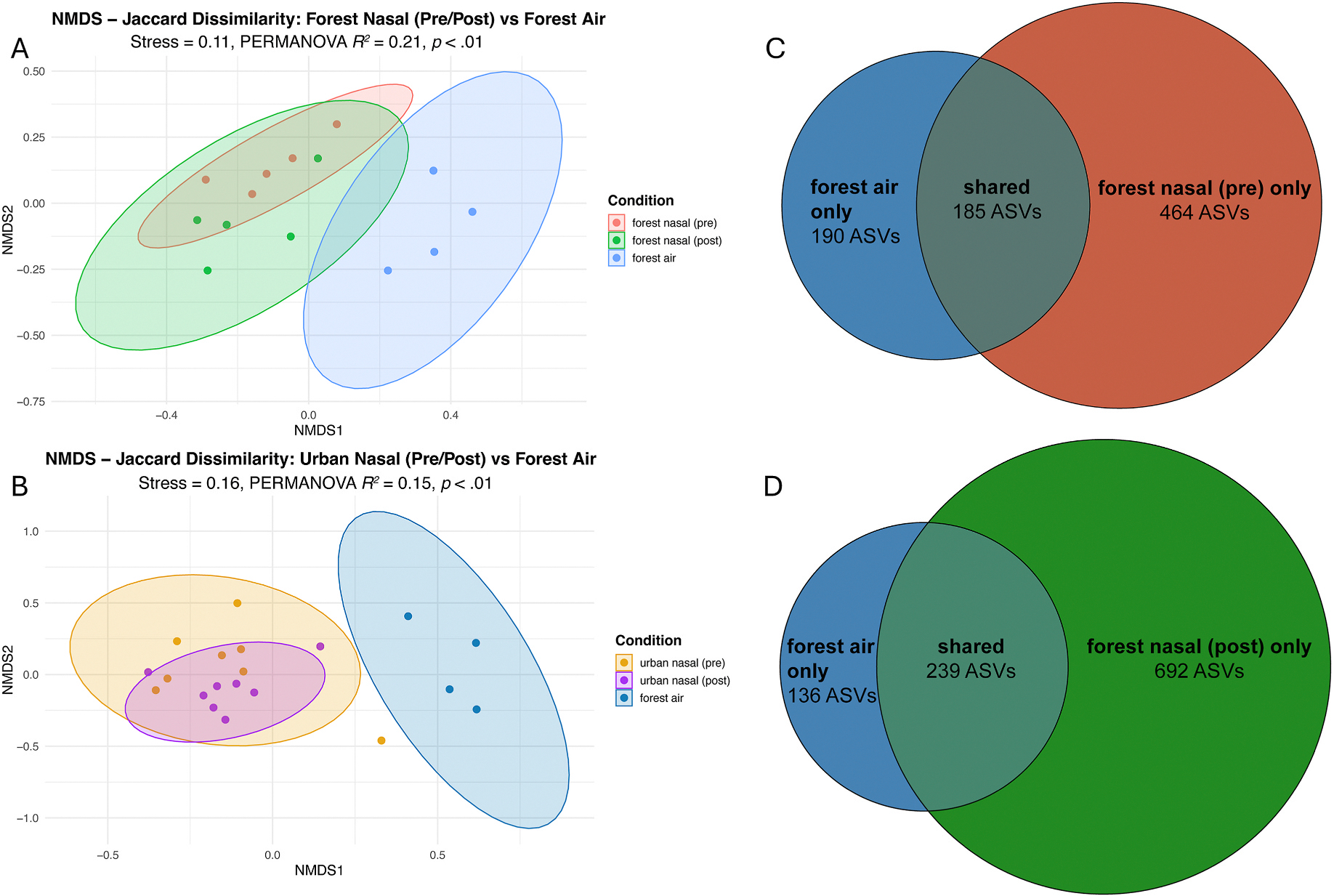
Compositional similarity between participants’ nasal microbiomes and the forest aerobiome. (A–B) NMDS ordination based on unweighted Jaccard dissimilarity comparing nasal microbiome samples from forest (A) and urban (B) groups pre- and post-exposure with forest aerobiome samples. Ellipses represent 95% confidence intervals around group centroids. (C–D) Venn diagrams showing ASV overlap between forest aerobiome samples and nasal microbiome samples from the forest group pre- (C) and post-exposure (D). The increase in shared ASVs from 185 (pre-exposure) to 239 (post-exposure) suggests potential microbial transfer from the forest aerobiome to the forest group’s nasal microbiomes, while the concurrent increase in forest group nasal-specific ASVs indicates exposure to additional environmental microbial reservoirs beyond airborne sources.

To explore whether the forest aerobiome could have influenced nasal microbiome composition, we compared nasal samples from each group to forest aerobiome samples using unweighted Jaccard dissimilarity and NMDS ordination (Fig. 4A and B). PERMANOVA revealed significant overall differences between nasal and forest aerobiome samples across all comparisons, all *p*s < .01. No significant changes in within-group dispersion were detected for either group’s nasal samples over time, all *p*s ≥ .48.

To further investigate potential links between airborne and nasal microbial communities, we assessed ASV overlap between forest group nasal samples and forest aerobiome samples. Pre-exposure, the overlap comprised 185 ASVs, which increased to 239 ASVs post-exposure (Fig. 4D). For comparison, the urban group exhibited the opposite trend, decreasing from 213 ASVs shared with the forest aerobiome pre-exposure to 195 ASVs post-exposure. While these findings do not constitute direct evidence that the forest aerobiome influenced nasal microbiome composition in the forest group, they are consistent with the possibility of microbial transfer from forest air to participants’ nasal passages. Interestingly, the number of ASVs unique to the forest group’s nasal samples—not detected in the air—also increased by 228 ASVs over time, indicating that observed changes in nasal bacterial community composition likely reflect additional routes of exposure from multiple environmental reservoirs (e.g., soil, plant surfaces). Future studies should examine these possibilities as well.

### 3.6. H5: Affective changes are associated with nasal bacterial community composition changes

**Fig. 5.**
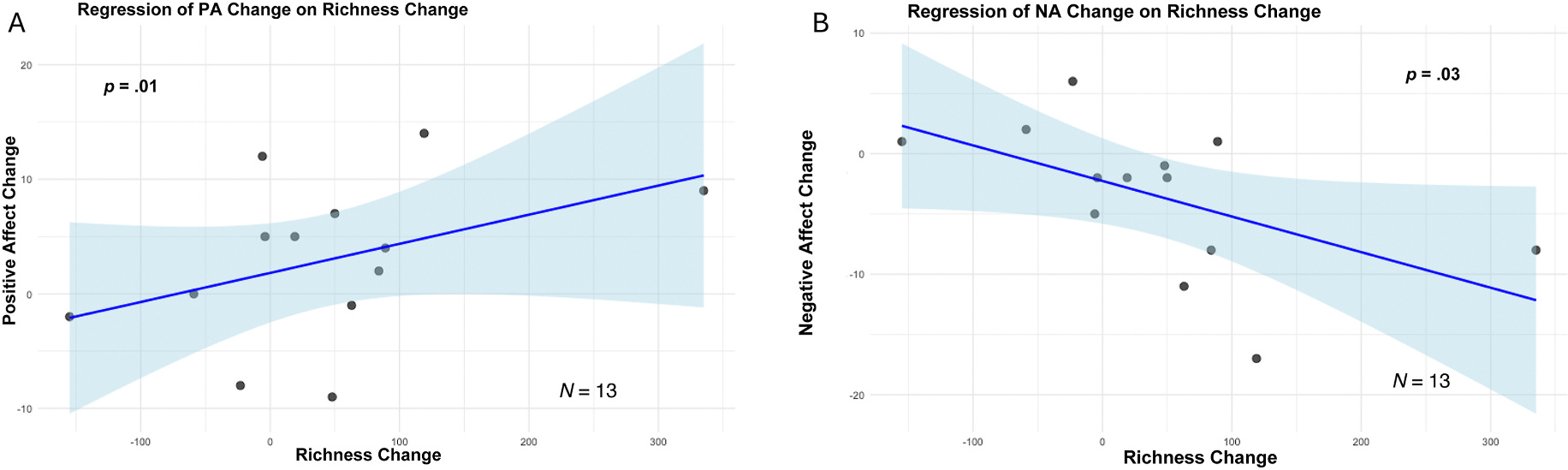
Associations between bacterial ASV richness change and affect change. Scatter plots depicting linear regression analyses of the relationships between nasal bacterial richness change and change in both positive (A) and negative affect (B) over time. Shaded areas represent 95% confidence intervals. Results indicate that, across all study participants, each additional one-ASV increase in mean nasal bacterial richness change over the exposure period was significantly associated with a 0.03 point greater increase in positive affect score and a 0.03 point greater decrease in negative affect score.

To examine whether changes in bacterial community composition were associated with changes in self-reported well-being, we conducted linear regression analyses with heteroskedasticity-robust standard errors. Plots of these regression models are displayed in Fig. 5.

Increased ASV richness was significantly associated with increased positive affect, *β* = 0.03, robust Wald 95% CI: 0.01–0.04, *p* = .01 (Fig. 5A), and decreased negative affect, *β* = −0.03, robust Wald 95% CI: −0.06 to −0.004, *p* = .03 (Fig. 5B). No clear association was observed between change in richness and change in rumination, and a weak trend was observed for the association between increased richness and increased mental well-being, *β* = 0.02, robust Wald 95% CI: 0.007–0.05, *p* = .14. Full model outputs are available in Table S7 in the Supplementary Materials. Based on Cook’s distance, no influential observations were detected for the positive affect model. For the negative affect model, two observations exceeded the common screening threshold of 4/*N*, which is not unexpected given the small sample size. Q-Q plots are provided in the Supplementary Materials (Figs. S6 and S7), and all results of this exploratory analysis should be interpreted with caution.

We also observed marginally significant associations between increased SDI and increased mental well-being, *β* = 9.00, robust Wald 95% CI: 0.17–17.82, *p* = .05, as well as decreased negative affect, *β* = −10.80, robust Wald 95% CI: −22.17 – 0.58, *p* = .06. Full model outputs are available in Table S8 in Supplementary Materials. Together, these findings support the possibility that increased bacterial α-diversity in the nasal passages may be associated with improvements in affect and mental well-being, particularly in terms of taxonomic richness, though these preliminary associations should be viewed as hypothesis-generating rather than evidence of causal psychotiotic effects given limited statistical power.

To further assess these relationships, we examined associations between changes in relative abundance of key taxa and change in well-being measures. We focused on four taxa enriched in the forest group—*Akkermansia, Bifidobacterium, Clostridium sensu stricto 1,* and *Muribaculaceae*—which have been previously linked to well-being improvements (Ding et al., 2021; Ma et al., 2022; M. Sharma et al., 2021; Volk et al., 2019). The full results of those regression models are provided in Table 2. Given the exploratory nature of this pilot study, these analyses were conducted without multiple-testing correction.

Increased relative abundance of *Akkermansia, Bifidobacterium,* and *Muribaculaceae* were significantly associated with increased positive affect. For example, results of the regression model suggest that, on average, each doubling of *Bifidobacterium* relative abundance was associated with a 0.80-point greater increase in positive affect score over time (95% robust Wald CI: 0.48 – 1.13 points). Additionally, increased relative abundance of *Akkermansia* and *Muribaculaceae* were associated with decreased negative affect. Together, these results suggest the possibility that changes in specific nasal taxa—some likely environmentally derived—may correspond with affective improvements following forest exposure. However, these relationships are preliminary and reported for the purpose of hypothesis-generation. Additional research is required to substantiate them and determine whether they reflect causal effects.

## 4. Discussion

This study used a natural experimental design to investigate associations of an 8-week forest vs. urban environment exposure with self-reported affect, rumination, and mental well-being; nasal microbiome composition; and correlations between these outcomes. We hypothesized that: (***H1***) forest vs. urban environment exposure would be associated with larger improvements in self-reported affect, rumination, and mental well-being measures, as well as with greater increases in nasal bacterial richness and SDI; (***H2***) overall nasal bacterial community composition would differ between groups post-exposure—but not pre-exposure—and forest environment-associated bacteria would be associated with forest group post-exposure samples; (***H3***) compositional overlap between the forest—but not urban—group’s microbiomes and the forest aerobiome would increase over the exposure period (***H4***) taxa associated with well-being benefits (e.g., immunoregulation, epithelial barrier maintenance, neuroprotection); would be enriched in the forest—but not urban—group post-exposure; and (***H5***) increases in nasal bacterial α-diversity and the relative abundance of taxa previously linked to well-being benefits would be associated with improvements in well-being measures.

In support of (***H1***), we found associations of forest vs. urban exposure with significant increases in positive affect, as well as decreases in negative affect and rumination. These findings align with previous research demonstrating associations of nature exposure with improved affect and emotion regulation (Berman et al., 2012; Bratman et al., 2015; Bratman et al., 2024; Hartig et al., 2014; White et al., 2021). We also observed higher bacterial richness in the forest vs. urban group post-exposure at a marginally statistically significant level, but lacking a significant group × time interaction, we cannot conclude that exposure conditions were differentially associated with richness change over time. Additionally, no corresponding pattern was evident for SDI. Together, these results provide supporting evidence for greater affective benefits of forest vs. urban environment exposure and limited evidence for greater increased nasal microbiome α-diversity.

In partial support of (***H2***), we found evidence for compositional—but not structural—differences in nasal bacterial communities across groups post-exposure. Unweighted analysis of taxonomic composition revealed a significant post-exposure difference between the forest and urban groups, whereas no difference was detected pre-exposure. However, weighted metrics, which account for relative abundance, showed no corresponding post-exposure differences between groups. Within-group analyses also revealed no significant compositional change from pre- to post-exposure in either group. Thus, while nasal bacterial community composition differed between groups post-exposure, we lack sufficient evidence to conclude that forest vs. urban environment exposure was associated with substantial post-exposure differences in community structure. The results also do not provide evidence that community composition or structure changed over time for either group. One possible explanation for these findings is that forest exposure may have facilitated the acquisition of low-abundance, forest-derived taxa—sufficient to produce detectable compositional differences between groups post-exposure, but not enough to meaningfully alter the underlying structure of the nasal microbiome. Future studies with larger samples and controlled exposure assessments are needed to determine whether and how microbial inputs from a forest environment contribute to shifts in the human nasal microbiome.

Indicator taxa analysis identified 24 ASVs significantly associated with the forest group post-exposure, all present at low relative abundance (<1%, on average). Most of these taxa are common in forest environmental niches, including soil, leaf surfaces, and freshwater sediments. For example, *Bryocella* and *Granulicella* belong to the phylum *Acidobacteriota*, which comprises 10–20% of the world’s soil bacteria and is also found in marine sediments (Barns et al., 1999; Conradie and Jacobs, 2021). These results support (***H2***), indicating that forest vs. urban environment exposure was associated with low-abundance, forest environment-associated bacteria in the nasal microbiome. However, it must be noted that their low relative abundance suggests they may represent transient environmental deposits with limited—if any—functional relevance.

In support of (***H3***), we found that the forest group’s nasal microbiomes shared more bacterial taxa with the forest aerobiome post-exposure compared to pre-exposure, while the urban group exhibited the opposite trend. Although these findings are consistent with the possibility that forest exposure introduced forest-associated bacteria into participants’ nasal microbiomes, we are not able to confirm direct deposition from the aerobiome with our study protocol. Moreover, the absence of statistically significant within-group changes in overall community composition or structure over time highlights the need for further research to clarify whether and how airborne microbes from a forest environment influence nasal microbiome composition.

In support of (***H4***), we found that the forest—but not urban—group exhibited enrichment over time of bacterial taxa previously associated with beneficial roles in health and well-being. Nineteen ASVs increased significantly in relative abundance within forest participants’ nasal microbiomes, including four genera—*Bifidobacterium, Akkermansia, Muribaculaceae, and Clostridium sensu stricto 1*—that have been linked in prior research to immunoregulatory, epithelial barrier-protective, and neuromodulatory functions.

Much of what is known about the biological roles of these genera comes from gut microbiome studies, where they have been examined far more extensively than in the nasal environment. For example, *Bifidobacterium* is a well-characterized genus typically associated with the human gut and extensively studied for its probiotic and psychobiotic potential (Allen et al., 2016; Groeger et al., 2020; Patterson et al., 2024; Savignac et al., 2014; Savignac et al., 2015; M. Sharma et al., 2021). Members of the genus produce tryptophan metabolites (e.g., indole-3-propionic acid (IPA) and indole-3-carbaldehyde (I3C)) that can bind to aryl hydrocarbon receptors in mucosal sites and trigger protective immunomodulatory responses (Alexeev et al., 2017; Fang et al., 2022). In murine models, these interactions in the gut are associated with improved cognition, synaptic plasticity, and reduced neuroinflammation (J. Sun et al., 2022). Certain *Bifidobacterium* species also produce ɣ-aminobutyric acid (GABA) (Altaib et al., 2022), a neurotransmitter linked to anxiolytic and anti-depressive effects (Kalueff and Nutt, 2007). Exopolysaccharides (EPS) from *Bifidobacterium* can suppress production of pro-inflammatory cytokines, such as TNF-α and IFN-ɣ (Fanning et al., 2012), and intranasal administration of bifidobacterial cell wall components is protective against inflammatory lung tissue damage in murine models of influenza (Groeger et al., 2020). While these mechanisms have been primarily observed in the gut, their immunomodulatory actions plausibly extend to other mucosal sites (Groeger et al., 2020), including the nasal passages; however, further investigation is required.

*Akkermansia* is a mucin-degrading genus typically abundant in healthy mucosa (Belzer and de Vos, 2012). Members produce short-chain fatty acids (SCFAs), like acetate and propionate (Si et al., 2022), which bind to G-protein-coupled receptors GPR41/FFAR3 and GPR43/FFAR2 on epithelial cells and initiate immunoregulatory cascades that help maintain balanced mucosal immune response, including in the airways (Lee et al., 2024). Murine models further indicate potential neuroprotective and mood-regulating effects. Oral administration of *A. muciniphila* improves intestinal barrier function, reduces neurotoxic peptide accumulation in the hippocampus, attenuates anxiety-like behaviors, and improves cognitive function (Ou et al., 2020). Similarly, supplementation with *Akkermansia* has been shown to improve depressive-like behavior, accompanied by inhibition of stress-induced increases in serum stress hormone concentration and restoration of dopamine and brain-derived neurotrophic factor (BDNF) expression in the hippocampus (Ding et al., 2021)—key neurochemical markers of mood regulation and stress resilience (Dunlop and Nemeroff, 2007; Martinowich and Lu, 2008). As GPR41/43 are also expressed on nasal airway epithelial cells (Imoto et al., 2018), enrichment of *Akkermansia* in the nasal microbiome may represent a plausible—though unverified—pathway through which forest exposure influences immunoregulatory and neuromodulatory pathways relevant to well-being.

*Muribaculaceae* can also degrade mucin and produce SCFAs like acetate, propionate, and succinate (Smith et al., 2019; Zhu et al., 2024), which may influence nasal mucosal immunity via the GPR41/43 pathways described previously. Its reduced abundance in the gut is linked to inflammatory bowel disease (IBD) (Volk et al., 2019). *Clostridium sensu stricto 1* is an SCFA producer associated with lower proinflammatory cytokine and gene expression levels in the colon (Ma et al., 2022). While these genera are less well-characterized, their SCFA-producing capacity and mucosal associations suggest they could contribute to immune balance and neuroimmune signaling in the nasal passages, pending further research.

In support of (***H5***), increased nasal bacterial richness was significantly associated with positive and negative affect (though not rumination and mental well-being) improvements across all participants. Moreover, increased relative abundance of each of the four taxa discussed above was significantly associated with affective improvements. While these are only preliminary associations and require cautious interpretation due to the exploratory nature of the study, they encourage further exploration of nasal microbiome-mediated pathways by which forest vs. urban environment exposure may impact well-being.

One hypothetical mechanism may be that forest vs. urban environment exposure selectively modulates the growth of certain bacterial taxa in the nasal passages—perhaps due to exposure to higher levels of biogenic volatile organic compounds (BVOCs) (Huang et al., 2019) or lower levels of air pollution (Mariani et al., 2018; Y. Sun et al., 2022)—resulting in increased relative abundance of taxa capable of modulating inflammatory response and increasing availability of well-being-promoting neuroactive compounds like serotonin (e.g., *Bifidobacterium, Akkermansia*) (Bae et al., 2022; Derrien et al., 2011; Ding et al., 2021; Fanning et al., 2012; Groeger et al., 2020; Yaghoubfar et al., 2020). These microbial changes could then both reduce local and systemic inflammation (Bae et al., 2022; Derrien et al., 2011; Groeger et al., 2013, 2020; Schiavi et al., 2016) and increase serotonin synthesis and release in the hippocampus (Misera et al., 2024; Yaghoubfar et al., 2020). Local inflammatory modulation could directly improve olfactory function by reducing swelling in the structures of the nasal passages (Doty, 2022), and systemic reductions in pro-inflammatory cytokines (e.g., IL-6, TNF-α) may induce a more regulated state of the hypothalamic-pituatary-adrenal (HPA) axis, characterized by an adaptive diurnal cortisol rhythm and improved serotonergic receptor function (Chrousos, 1995; Watson and Mackin, 2006).

By this theoretical mechanism, the final physiological outcomes could include reduced systemic pro-inflammatory biomarkers, improved olfactory function, regulated diurnal cortisol rhythms, and increased availability of serotonin in the hippocampus concurrent with improved serotonergic receptor function. Psychological benefits could include reduced perceived stress and negative affect (Alenina and Klempin, 2015; Knight et al., 2021), as well as increased positive affect (Zuccarella-Hackl et al., 2023) and improved cognitive function (Marsland et al., 2015). We emphasize, however, that while there is evidence for the plausibility of each of these mechanistic steps in the literature, the complete pathway is speculative, and substantial additional research is necessary to evaluate its validity. We include it as an example of one of many potential mechanisms to explore in future research.

Despite the intriguing nature of these findings, we acknowledge several key limitations of this exploratory pilot study. First, the small sample size (*N* = 13) substantially limited statistical power, and replication in larger cohorts will be necessary to validate these results. Future studies should include an *a priori* power analysis to determine an appropriate sample size for detecting effects of interest. Second, the natural experimental design did not allow for random assignment of participants, introducing potential preselection bias and unmeasured confounding. Although we excluded individuals with diagnosed psychiatric conditions, recent illnesses, or who took medications that could affect the nasal microbiome, and no significant baseline group differences were detected in age, sex at birth, race/ethnicity, diet, alcohol use, or our outcome variables, other pre-existing factors (e.g., cohabitation with pets, temperament) may still have influenced measured outcomes. In addition, several unmeasured factors that could plausibly have differed between the forest and urban settings during the exposure period may have contributed to the psychological improvements observed in the forest group independent of, or in addition to, nasal microbiome changes. These include academic load, physical activity levels, sleep quality, social interactions, dietary changes, general sense of being away, weather conditions, and air pollution. Future studies should incorporate design elements (e.g., GPS-based monitoring of participants, wearable heart-rate or accelerometry-based devices, diet tracking, air pollution monitoring at study sites) and analytic strategies to account for these sources of variability. Third, regression analyses of well-being change on individual taxa relative abundance change were performed without correction for multiple-testing due to the exploratory nature of the study. However, this increases the risk of type I error. As emphasized throughout, results are preliminary and intended to be hypothesis-generating rather than confirmatory. Fourth, because non-sterile cones were used for air sample collection, the aerobiome results should be interpreted with extreme caution, as contamination during manufacturing or packaging cannot be ruled out. Although statistical contaminant identification methods flagged no ASVs in forest air samples as contaminants, this is likely the result of low biomass, low statistical power, or substantial compositional overlap between samples and the control blank. Finally, to evaluate the efficacy of self-collection procedures, participants collected their own nasal swab samples under supervision of the study team. Detailed instructions were provided to each participant prior to sample collection, and unsatisfactory samples (e.g., touched the exterior of the nostril) were discarded, but minor variation in swab placement within the nose, or pressure applied to the interior walls, could impact bacterial community composition profiles.

Future research should seek to replicate and extend these preliminary findings using randomized designs, larger samples, and comprehensive multi-omic analyses (e.g., metagenomics, transcriptomics, metabolomics) to examine functional shifts and mechanisms. Additional priorities could include determining minimum exposure duration and persistence of changes, incorporating local and systemic inflammatory biomarkers, and parsing individual environmental components (e.g., air pollution, aerobiome, BVOCs) that may drive changes in both well-being and the nasal microbiome. Notably, recent work reexamining the ‘open-air factor’ (OAF), first described in the 1960s (Druett and May, 1968), highlights antimicrobial properties of outside vs. inside air that remain incompletely understood (Cox et al., 2021). It is possible that the OAF or BVOCs like pinenes, which are known to be antimicrobial (Silva et al., 2012), initially deplete urban residents’ nasal microbiomes during forest exposure, opening ecological niches to colonization by forest-adapted microorganisms. Identifying such “active ingredients” of nature could inform public health efforts to enhance microbial exposures in urban settings for the promotion of human well-being and bolster the rationale for conserving large areas of nature outside of cities as determinants of human health and well-being (Matthews et al., 2024).

## 5. Conclusion

This exploratory pilot study provides preliminary evidence that 8-week exposure to a forest vs. urban environment is associated with increases in positive affect and decreases in negative affect and rumination, accompanied by compositional changes in the nasal microbiome that correlate with well-being improvements. The forest group exhibited higher post-exposure bacterial richness than the urban group at a marginally statistically significant level, as well as significant enrichment of genera such as *Bifidobacterium* and *Akkermansia*, which have been linked in prior research to immunoregulatory and neuromodulatory functions relevant to well-being. Moreover, increased relative abundance of these and other taxa correlated with affective improvements, suggesting that microbiome-mediated mechanisms warrant further investigation. Although the small sample size and natural experimental design constrain causal inferences, these findings highlight the nasal microbiome as a promising and underexplored pathway through which nature exposure may influence human well-being.

## Supplementary Material

MMC1

## Figures and Tables

**Table 1:** Participant demographics.

Characteristic	Urban	Forest

**Total participants (n)**	8	5
**Mean age (years)**	22	21
**Sex at birth**		
**Male (n)**	2	1
**Female (n)**	6	4
**Race/ethnicity**		
**Asian (n)**	4	0
**Hispanic or Latino (n)**	1	1
**White (n)**	3	4

**Table 2:** Associations between relative abundance changes in key nasal bacterial taxa and psychological measures of affect, rumination, and mental well-being.

Taxon	Well-being measure	*β*	Robust SE	95% CI		*p*-value
				Lower	Upper	

*Akkermansia*	Positive affect (PANAS)	0.69	0.19	0.27	1.12	**< .01**
	Negative affect (PANAS)	−0.28	0.10	−0.49	−0.06	**.02**
	Rumination (RRQ)	0.006	0.02	−0.04	0.05	.77
	Mental well-being (WEMWBS)	0.68	0.40	−0.20	1.56	.12
						
*Bifidobacterium*	Positive affect (PANAS)	0.80	0.15	0.48	1.13	**< .01**
	Negative affect (PANAS)	−0.24	0.27	−0.83	0.35	.39
	Rumination (RRQ)	−0.02	0.02	−0.05	0.02	.32
	Mental well-being (WEMWBS)	0.24	0.27	−0.35	0.82	.39
						
*Clostridium sensu stricto 1*	Positive affect (PANAS)	0.42	0.17	0.04	0.80	**.03**
	Negative affect (PANAS)	−0.02	0.22	−0.52	0.47	.92
	Rumination (RRQ)	0.005	0.02	−0.03	0.04	.79
	Mental well-being (WEMWBS)	0.11	0.22	−0.37	0.59	.63
						
*Muribaculaceae*	Positive affect (PANAS)	0.50	0.16	0.15	0.85	**.01**
	Negative affect (PANAS)	−0.40	0.13	−0.68	−0.12	**.01**
	Rumination (RRQ)	−0.02	0.01	−0.04	0.01	.25
	Mental well-being (WEMWBS)	0.32	0.25	−0.23	0.87	.23
						
*Gemmatimonas*	Positive affect (PANAS)	0.32	0.48	−0.73	1.37	.52
	Negative affect (PANAS)	−0.74	0.37	−1.55	0.06	.07
	Rumination (RRQ)	−0.03	0.03	−0.10	0.04	.33
	Mental well-being (WEMWBS)	0.28	0.49	−0.80	1.37	.58

Results from regression analyses examining the relationship between log_2_ fold change in five bacterial genera and changes in positive affect, negative affect, rumination, and mental well-being across all study participants. The *p*-values provided are uncorrected for multiple testing. ***β*** = expected mean difference in survey score change; **Robust SE** = robust Wald standard error; **CI** = confidence interval.

## Data Availability

Data will be made available on request.

## Appendix A. Supplementary data

Supplementary data to this article can be found online at https://doi.org/10.1016/j.envres.2025.123582.

## References

[R1] Alenina N, Klempin F, 2015. The role of serotonin in adult hippocampal neurogenesis. Behav. Brain Res. 277, 49–57. 10.1016/j.bbr.2014.07.038. 25125239

[R2] Alexeev EE, Lanis JM, Kao DJ, Battista KD, Kelly CJ, Campbell EL, Kominsky DJ, Colgan SP, 2017. Microbiota-derived tryptophan metabolites provide a novel pathway for regulation of mucosal barrier function. FASEB J. 31 (S1), 469.469. 10.1096/fasebj.31.1_supplement.469.9, 469.469.27623929

[R3] Allen AP, Hutch W, Borre YE, Kennedy PJ, Temko A, Boylan G, Murphy E, Cryan JF, Dinan TG, Clarke G, 2016. Bifidobacterium longum 1714 as a translational psychobiotic: modulation of stress, electrophysiology and neurocognition in healthy volunteers. Transl. Psychiatry 6 (11), e939. 10.1038/tp.2016.191 e939. 27801892 PMC5314114

[R4] Altaib H, Kozakai T, Badr Y, Nakao H, El-Nouby MAM, Yanase E, Nomura I, Suzuki T, 2022. Cell factory for γ-aminobutyric acid (GABA) production using Bifidobacterium adolescentis. Microb. Cell Fact. 21 (1), 33. 10.1186/s12934-021-01729-6. 35255900 PMC8903651

[R5] Andersen L, Corazon SS, Stigsdotter UK, 2021. Nature exposure and its effects on immune system functioning: a systematic review. Int. J. Environ. Res. Publ. Health 18 (4), 1416. 10.3390/ijerph18041416. PMC7913501 33546397

[R6] Asri AK, Liu T, Tsai H-J, Lee H-Y, Pan W-C, Wu C-D, Wang J-Y, 2023. Residential greenness and air pollution’s association with nasal microbiota among asthmatic children. Environ. Res. 219, 115095. 10.1016/j.envres.2022.115095. 36535395

[R7] Bae M, Cassilly CD, Liu X, Park S-M, Tusi BK, Chen X, Kwon J, Filipčík P, Bolze AS, Liu Z, Vlamakis H, Graham DB, Buhrlage SJ, Xavier RJ, Clardy J, 2022. Akkermansia muciniphila phospholipid induces homeostatic immune responses. Nature 608 (7921), 168–173. 10.1038/s41586-022-04985-7. 35896748 PMC9328018

[R8] Barns SM, Takala SL, Kuske CR, 1999. Wide distribution and diversity of members of the bacterial kingdom Acidobacterium in the environment. Appl. Environ. Microbiol. 65 (4), 1731–1737. 10.1128/AEM.65.4.1731-1737.1999. 10103274 PMC91244

[R9] Bates D, Mächler M, Bolker B, Walker S, 2015. Fitting linear mixed-effects models using lme4. J. Stat. Software 67 (1), 1–48. 10.18637/jss.v067.i01.

[R10] Belzer C, de Vos WM, 2012. Microbes inside—from diversity to function: the case of Akkermansia. ISME J. 6 (8), 1449–1458. 10.1038/ismej.2012.6. 22437156 PMC3401025

[R11] Benjamini Y, Hochberg Y, 1995. Controlling the false discovery rate: a practical and powerful approach to multiple testing. J. Roy. Stat. Soc. B 57 (1), 289–300. 10.1111/j.2517-6161.1995.tb02031.x.

[R12] Berman MG, Kross E, Krpan KM, Askren MK, Burson A, Deldin PJ, Kaplan S, Sherdell L, Gotlib IH, Jonides J, 2012. Interacting with nature improves cognition and affect for individuals with depression. J. Affect. Disord. 140 (3), 300–305. 10.1016/j.jad.2012.03.012. 22464936 PMC3393816

[R13] Biswas K, Wagner Mackenzie B, Ballauf C, Draf J, Douglas RG, Hummel T, 2020. Loss of bacterial diversity in the sinuses is associated with lower smell discrimination scores. Sci. Rep. 10 (1), 16422. 10.1038/s41598-020-73396-3. 33009469 PMC7532173

[R14] Bolyen E, Rideout JR, Dillon MR, Bokulich NA, Abnet CC, Al-Ghalith GA, Alexander H, Alm EJ, Arumugam M, Asnicar F, Bai Y, Bisanz JE, Bittinger K, Brejnrod A, Brislawn CJ, Brown CT, Callahan BJ, Caraballo-Rodríguez AM, Chase J, Caporaso JG, 2019. Reproducible, interactive, scalable and extensible microbiome data science using QIIME 2. Nat. Biotechnol. 37 (8), 852–857. 10.1038/s41587-019-0209-9. 31341288 PMC7015180

[R15] Bossé JT, Bujold AR, Li L, 2022. Actinobacillus. In: Pathogenesis of Bacterial Infections in Animals, pp. 262–289. 10.1002/9781119754862.ch12.

[R16] Bowe P, 2017. The early development of garden making c. 3000–c. 2000 BCE. Stud. Hist. Gard. Des. Landsc. 37 (3), 231–241. 10.1080/14601176.2017.1318579.

[R17] Brahimi S, Cadoret F, Founier PE, Moal V, Raoult D, 2017. ‘Peptoniphilus urinimassiliensis’ sp. nov., a new bacterial species isolated from a human urine sample after de novo kidney transplantation. New Microbes New Infect 16, 49–50. 10.1016/j.nmni.2017.01.001. 28203376 PMC5294733

[R18] Brame JE, Liddicoat C, Abbott CA, Breed MF, 2021. The potential of outdoor environments to supply beneficial butyrate-producing bacteria to humans. Sci. Total Environ. 777, 146063. 10.1016/j.scitotenv.2021.146063. 33684759

[R19] Brame JE, Warbrick I, Heke D, Liddicoat C, Breed MF, 2024. Short-term passive greenspace exposures have little effect on nasal microbiomes: a cross-over exposure study of a Māori cohort. Environ. Res. 252, 118814. 10.1016/j.envres.2024.118814. 38555095

[R20] Bratman GN, Anderson CB, Berman MG, Cochran B, De Vries S, Flanders J, Folke C, Frumkin H, Gross JJ, Hartig T, Kahn PH, Kuo M, Lawler JJ, Levin PS, Lindahl T, Meyer-Lindenberg A, Mitchell R, Ouyang Z, Roe J, Daily GC, 2019. Nature and mental health: an ecosystem service perspective. Sci. Adv. 5 (7). 10.1126/sciadv.aax0903 eaax0903. PMC6656547 31355340

[R21] Bratman GN, Bembibre C, Daily GC, Doty RL, Hummel T, Jacobs LF, Kahn PH, Lashus C, Majid A, Miller JD, Oleszkiewicz A, Olvera-Alvarez H, Parma V, Riederer AM, Sieber NL, Williams J, Xiao J, Yu C-P, Spengler JD, 2024a. Nature and human well-being: the olfactory pathway. Sci. Adv. 10 (20). 10.1126/sciadv.adn3028 eadn3028. PMC11809653 38748806

[R22] Bratman GN, Daily GC, Levy BJ, Gross JJ, 2015. The benefits of nature experience: improved affect and cognition. Landsc. Urban Plann. 138, 41–50. 10.1016/j.landurbplan.2015.02.005.

[R23] Bratman GN, Mehta A, Olvera-Alvarez H, Spink KM, Levy C, White MP, Kubzansky LD, Gross JJ, 2024b. Associations of nature contact with emotional ill-being and well-being: the role of emotion regulation. Cognit. Emot. 38 (5), 748–767. 10.1080/02699931.2024.2316199. 38362747

[R24] Callahan BJ, McMurdie PJ, Rosen MJ, Han AW, Johnson AJA, Holmes SP, 2016. DADA2: High-resolution sample inference from Illumina amplicon data. Nat. Methods 13 (7), 581–583. 10.1038/nmeth.3869. 27214047 PMC4927377

[R25] Chen YT, Williamson BD, Okonek T, Wolock CJ, Spieker AJ, Wai TYH, Hughes JP, Emerson SS, Willis AD, 2022. Rigr: regression, inference, and general data analysis tools in R. J. Open Source Softw. 7 (80), 4847.

[R26] Cheng L-H, Liu Y-W, Wu C-C, Wang S, Tsai Y-C, 2019. Psychobiotics in mental health, neurodegenerative and neurodevelopmental disorders. J. Food Drug Anal. 27 (3), 632–648. 10.1016/j.jfda.2019.01.002. 31324280 PMC9307042

[R27] Cho D-Y, Skinner D, Hunter RC, Weeks C, Lim DJ, Thompson H, Walz CR, Zhang S, Grayson JW, Swords WE, Rowe SM, Woodworth BA, 2020. Contribution of short chain fatty acids to the growth of Pseudomonas aeruginosa in rhinosinusitis. Front. Cell. Infect. Microbiol. 10. 10.3389/fcimb.2020.00412. PMC7431473 32850504

[R28] Chrousos GP, 1995. The hypothalamic–pituitary–adrenal axis and immune-mediated inflammation. N. Engl. J. Med. 332 (20), 1351–1363. 10.1056/NEJM199505183322008. 7715646

[R29] Conradie TA, Jacobs K, 2021. Distribution patterns of Acidobacteriota in different fynbos soils. PLoS One 16 (3), e0248913. 10.1371/journal.pone.0248913. 33750980 PMC7984625

[R30] Cox RA, Ammann M, Crowley JN, Griffiths PT, Herrmann H, Hoffmann EH, Jenkin ME, McNeill VF, Mellouki A, Penkett CJ, Tilgner A, Wallington TJ, 2021. Opinion: the germicidal effect of ambient air (open-air factor) revisited. Atmos. Chem. Phys. 21 (17), 13011–13018. 10.5194/acp-21-13011-2021.

[R31] Dalile B, Van Oudenhove L, Vervliet B, Verbeke K, 2019. The role of short-chain fatty acids in microbiota–gut–brain communication. Nat. Rev. Gastroenterol. Hepatol. 16 (8), 461–478. 10.1038/s41575-019-0157-3. 31123355

[R32] Dalton KR, Louis LM, Fandiño-Del-Rio M, Rule AM, Pool W, Randolph K, Thomas S, Davis MF, Quirós-Alcalá L, 2022. Microbiome alterations from volatile organic compounds (VOC) exposures among workers in salons primarily serving women of color. Environ. Res. 214, 114125. 10.1016/j.envres.2022.114125. 35987373 PMC11316258

[R33] Davis NM, Proctor DM, Holmes SP, Relman DA, Callahan BJ, 2018. Simple statistical identification and removal of contaminant sequences in marker-gene and metagenomics data. Microbiome 6 (1), 226. 10.1186/s40168-018-0605-2. 30558668 PMC6298009

[R34] Derrien M, van Baarlen P, Hooiveld G, Norin E, Muller M, de Vos W, 2011. Modulation of mucosal immune response, tolerance, and proliferation in mice colonized by the mucin-degrader Akkermansia muciniphila [Original Research]. Front. Microbiol. 2. 10.3389/fmicb.2011.00166, 2011. PMC3153965 21904534

[R35] Dikeçligil GN, Gottfried JA, 2024. What does the human olfactory system do, and how does it do it? Annu. Rev. Psychol. 75, 155–181. 10.1146/annurev-psych-042023-101155. Volume 75, 2024. 37788573 PMC12931680

[R36] Dinan TG, Stanton C, Cryan JF, 2013. Psychobiotics: a novel class of psychotropic. Biol. Psychiatry 74 (10), 720–726. 10.1016/j.biopsych.2013.05.001. 23759244

[R37] Ding Y, Bu F, Chen T, Shi G, Yuan X, Feng Z, Duan Z, Wang R, Zhang S, Wang Q, Zhou J, Chen Y, 2021. A next-generation probiotic: akkermansia muciniphila ameliorates chronic stress–induced depressive-like behavior in mice by regulating gut microbiota and metabolites. Appl. Microbiol. Biotechnol. 105 (21), 8411–8426. 10.1007/s00253-021-11622-2. 34617139

[R38] Doty RL, 2022. Olfactory dysfunction in COVID-19: pathology and long-term implications for brain health. Trends Mol. Med. 28 (9), 781–794. 10.1016/j.molmed.2022.06.005. 35810128 PMC9212891

[R39] Druett HA, May KR, 1968. Unstable germicidal pollutant in rural air. Nature 220 (5165), 395–396. 10.1038/220395a0. 4879332

[R40] Dunlop BW, Nemeroff CB, 2007. The role of dopamine in the pathophysiology of depression. Arch. Gen. Psychiatry 64 (3), 327–337. 10.1001/archpsyc.64.3.327. 17339521

[R41] Elliott LR, Pasanen T, White MP, Wheeler BW, Grellier J, Cirach M, Bratman GN, van den Bosch M, Roiko A, Ojala A, 2023. Nature contact and general health: testing multiple serial mediation pathways with data from adults in 18 countries. Environ. Int. 178, 108077. 37413929 10.1016/j.envint.2023.108077

[R42] Fang Z, Tong P, Lingzhi L, Hongchao W, Jinlin Z, Hao Z, Jianxin Z, Wei C, Lu W, 2022. Bifidobacterium longum mediated tryptophan metabolism to improve atopic dermatitis via the gut-skin axis. Gut Microbes 14 (1), 2044723. 10.1080/19490976.2022.2044723. 35239463 PMC8903757

[R43] Fanning S, Hall LJ, Cronin M, Zomer A, MacSharry J, Goulding D, O’Connell Motherway M, Shanahan F, Nally K, Dougan G, van Sinderen D, 2012. Bifidobacterial surface-exopolysaccharide facilitates commensal-host interaction through immune modulation and pathogen protection. Proc. Natl. Acad. Sci. 109 (6), 2108–2113. 10.1073/pnas.1115621109. 22308390 PMC3277520

[R44] Fayet-Moore F, Robinson SR, 2024. A breath of fresh air: perspectives on inhaled nutrients and bacteria to improve human health. Adv. Nutr. 15 (12), 100333. 10.1016/j.advnut.2024.100333. 39486624 PMC11626012

[R45] Figueiredo G, Gomes M, Covas C, Mendo S, Caetano T, 2022. The unexplored wealth of microbial secondary metabolites: the Sphingobacteriaceae case study. Microb. Ecol. 83 (2), 470–481. 10.1007/s00248-021-01762-3. 33987687

[R46] Garrett JK, White MP, Elliott LR, Grellier J, Bell S, Bratman GN, Economou T, Gascon M, Lõhmus M, Nieuwenhuijsen M, 2023. Applying an ecosystem services framework on nature and mental health to recreational blue space visits across 18 countries. Sci. Rep. 13 (1), 2209. 36878999 10.1038/s41598-023-28544-w PMC9988977

[R47] Gisler A, Korten I, de Hoogh K, Vienneau D, Frey U, Decrue F, Gorlanova O, Soti A, Hilty M, Latzin P, Usemann J, 2021. Associations of air pollution and greenness with the nasal microbiota of healthy infants: a longitudinal study. Environ. Res. 202, 111633. 10.1016/j.envres.2021.111633. 34256075

[R48] Groeger D, O’Mahony L, Murphy EF, Bourke JF, Dinan TG, Kiely B, Shanahan F, Quigley EMM, 2013. Bifidobacterium infantis 35624 modulates host inflammatory processes beyond the gut. Gut Microbes 4 (4), 325–339. 10.4161/gmic.25487. 23842110 PMC3744517

[R49] Groeger D, Schiavi E, Grant R, Kurnik-Łucka M, Michalovich D, Williamson R, Beinke S, Kiely B, Akdis CA, Hessel EM, Shanahan F, O’ Mahony L, 2020. Intranasal *Bifidobacterium longum* protects against viral-induced lung inflammation and injury in a murine model of lethal influenza infection. EBioMedicine 60. 10.1016/j.ebiom.2020.102981. PMC7495089 32927273

[R50] Harris PA, Taylor R, Minor BL, Elliott V, Fernandez M, O’Neal L, McLeod L, Delacqua G, Delacqua F, Kirby J, Duda SN, 2019. The REDCap consortium: building an international community of software platform partners. J. Biomed. Inf. 95, 103208. 10.1016/j.jbi.2019.103208. PMC7254481 31078660

[R51] Harris PA, Taylor R, Thielke R, Payne J, Gonzalez N, Conde JG, 2009. Research electronic data capture (REDCap)—A metadata-driven methodology and workflow process for providing translational research informatics support. J. Biomed. Inf. 42 (2), 377–381. 10.1016/j.jbi.2008.08.010. PMC2700030 18929686

[R52] Hartig T, Mitchell R, de Vries S, Frumkin H, 2014. Nature and health. Annu. Rev. Publ. Health 35 (35), 207. 10.1146/annurev-publhealth-032013-182443. 24387090

[R53] Hashimoto K, 2023. Emerging role of the host microbiome in neuropsychiatric disorders: overview and future directions. Mol. Psychiatr. 28 (9), 3625–3637. 10.1038/s41380-023-02287-6. PMC10730413 37845499

[R54] Hoggard M, Biswas K, Zoing M, Wagner Mackenzie B, Taylor MW, Douglas RG, 2017. Evidence of microbiota dysbiosis in chronic rhinosinusitis. Int. Forum Allergy Rhinol. 7 (3), 230–239. 10.1002/alr.21871. 27879060

[R55] Hoggard M, Nocera A, Biswas K, Taylor MW, Douglas RG, Bleier BS, 2018. The sinonasal microbiota, neural signaling, and depression in chronic rhinosinusitis. Int. Forum Allergy Rhinol. 8 (3), 394–405. 10.1002/alr.22074. 29278464

[R56] Huang AC, Jiang T, Liu Y-X, Bai Y-C, Reed J, Qu B, Goossens A, Nützmann H-W, Bai Y, Osbourn A, 2019. A specialized metabolic network selectively modulates *Arabidopsis* root microbiota. Science 364 (6440). 10.1126/science.aau6389 eaau6389. 31073042

[R57] Imoto Y, Kato A, Takabayashi T, Sakashita M, Norton JE, Suh LA, Carter RG, Weibman AR, Hulse KE, Stevens W, Harris KE, Peters AT, Grammer LC, Tan BK, Welch K, Conley DB, Kern RC, Fujieda S, Schleimer RP, 2018. Short-chain fatty acids induce tissue plasminogen activator in airway epithelial cells via GPR41&43. Clin. Exp. Allergy 48 (5), 544–554. 10.1111/cea.13119. 29431874 PMC5987538

[R58] Kalueff AV, Nutt DJ, 2007. Role of GABA in anxiety and depression. Depress. Anxiety 24 (7), 495–517. 10.1002/da.20262. 17117412

[R59] Kaplan S, 1995. The restorative benefits of nature: toward an integrative framework. J. Environ. Psychol. 15 (3), 169–182. 10.1016/0272-4944(95)90001-2.

[R60] Klindworth A, Pruesse E, Schweer T, Peplies J, Quast C, Horn M, Glöckner FO, 2013. Evaluation of general 16S ribosomal RNA gene PCR primers for classical and next-generation sequencing-based diversity studies. Nucleic Acids Res. 41 (1), e1. 10.1093/nar/gks808 e1. 22933715 PMC3592464

[R61] Knight EL, Jiang Y, Rodriguez-Stanley J, Almeida DM, Engeland CG, Zilioli S, 2021. Perceived stress is linked to heightened biomarkers of inflammation via diurnal cortisol in a national sample of adults. Brain Behav. Immun. 93, 206–213. 10.1016/j.bbi.2021.01.015. 33515741 PMC8274563

[R62] Konovalovas A, Armalytė J, Klimkaitė L, Liveikis T, Jonaitytė B, Danila E, Bironaitė D, Mieliauskaitė D, Bagdonas E, Aldonytė R, 2024. Human nasal microbiota shifts in healthy and chronic respiratory disease conditions. BMC Microbiol. 24 (1), 150. 10.1186/s12866-024-03294-5. 38678223 PMC11055347

[R63] Koskinen K, Reichert JL, Hoier S, Schachenreiter J, Duller S, Moissl-Eichinger C, Schöpf V, 2018. The nasal microbiome mirrors and potentially shapes olfactory function. Sci. Rep. 8 (1), 1296. 10.1038/s41598-018-19438-3. 29358754 PMC5778015

[R64] Kraemer JG, Aebi S, Hilty M, Oppliger A, 2021. Nasal microbiota composition dynamics after occupational change in animal farmers suggest major shifts. Sci. Total Environ. 782, 146842. 10.1016/j.scitotenv.2021.146842. 33838360

[R65] Kraemer JG, Ramette A, Aebi S, Oppliger A, Hilty M, 2018. Influence of pig farming on the human nasal microbiota: key role of airborne microbial communities. Appl. Environ. Microbiol. 84 (6). 10.1128/AEM.02470-17 e02470–02417. 29330190 PMC5835734

[R66] Kumpitsch C, Koskinen K, Schopf V, Moissl-Eichinger C, 2019. The microbiome of the upper respiratory tract in health and disease. BMC Biol 17 (1). 10.1186/s12915-019-0703-z. PMC6836414 31699101

[R67] Kuo M, 2015. How might contact with nature promote human health? Promising mechanisms and a possible central pathway [Mini Review]. Front. Psychol. 6. 10.3389/fpsyg.2015.01093. PMC4548093 26379564

[R68] Kuznetsova A, Brockhoff PB, Christensen RHB, 2017. lmerTest package: tests in linear mixed effects models. J. Stat. Software 82 (13), 1–26. 10.18637/jss.v082.i13.

[R69] Larsson J, 2024. Eulerr: Area-Proportional Euler and Venn Diagrams with Ellipses. CRAN. In (Version R package version 7.0.2). https://cran.r-project.org/package=eulerr.

[R70] Lazarini F, Roze E, Lannuzel A, Lledo PM, 2022. The microbiome-nose-brain axis in health and disease. Trends Neurosci. 45 (10), 718–721. 10.1016/j.tins.2022.08.003. 36055893

[R71] Lee D-H, Kim M-T, Han J-H, 2024. GPR41 and GPR43: from development to metabolic regulation. Biomed. Pharmacother. 175, 116735. 10.1016/j.biopha.2024.116735. 38744220

[R72] Li Q, 2010. Effect of forest bathing trips on human immune function. Environ. Health Prev. Med. 15 (1), 9–17. 10.1007/s12199-008-0068-3. 19568839 PMC2793341

[R73] Liddicoat C, Sydnor H, Cando-Dumancela C, Dresken R, Liu J, Gellie NJC, Mills JG, Young JM, Weyrich LS, Hutchinson MR, Weinstein P, Breed MF, 2020. Naturally-diverse airborne environmental microbial exposures modulate the gut microbiome and may provide anxiolytic benefits in mice. Sci. Total Environ. 701, 134684. 10.1016/j.scitotenv.2019.134684. 31704402

[R74] Lou H, Liu X, Liu P, 2023. Mechanism and implications of pro-nature physical activity in antagonizing psychological stress: the key role of microbial-gut-brain axis. Front. Psychol. 14, 1143827. 37560094 10.3389/fpsyg.2023.1143827 PMC10408457

[R75] Love MI, Huber W, Anders S, 2014. Moderated estimation of fold change and dispersion for RNA-seq data with DESeq2. Genome Biol. 15 (12), 550. 10.1186/s13059-014-0550-8. 25516281 PMC4302049

[R76] Ma H, Dong Z, Zhang X, Liu C, Liu Z, Zhou X, He J, Zhang S, 2024. Airway bacterial microbiome signatures correlate with occupational pneumoconiosis progression. Ecotoxicol. Environ. Saf. 284, 116875. 10.1016/j.ecoenv.2024.116875. 39142114

[R77] Ma L, Shen Q, Lyu W, lv L., Wang W., Yu M., Yang H, Tao S., Xiao Y., 2022. Clostridium butyricum and its derived extracellular vesicles modulate gut homeostasis and ameliorate acute experimental colitis. Microbiol. Spectr. 10 (4). 10.1128/spectrum.01368-22 e01368-01322. PMC9431305 35762770

[R78] Maki JJ, Howard M, Connelly S, Pettengill MA, Hardy DJ, Cameron A, 2023. Species delineation and comparative genomics within the Campylobacter ureolyticus complex. J. Clin. Microbiol. 61 (5). 10.1128/jcm.00046-23 e00046–00023. 37129508 PMC10204631

[R79] Marcus CC, Barnes M, 1999. Healing Gardens: Therapeutic Benefits and Design Recommendations, 4. John Wiley & Sons.

[R80] Mariani J, Favero C, Spinazzè A, Cavallo DM, Carugno M, Motta V, Bonzini M, Cattaneo A, Pesatori AC, Bollati V, 2018. Short-term particulate matter exposure influences nasal microbiota in a population of healthy subjects. Environ. Res. 162, 119–126. 10.1016/j.envres.2017.12.016. 29291434

[R81] Marselle MR, Hartig T, Cox DTC, De Bell S, Knapp S, Lindley S, Triguero-Mas M, Böhning-Gaese K, Braubach M, Cook PA, De Vries S, Heintz-Buschart A, Hofmann M, Irvine KN, Kabisch N, Kolek F, Kraemer R, Markevych I, Martens D, Bonn A, 2021. Pathways linking biodiversity to human health: a conceptual framework. Environ. Int. 150, 106420. 10.1016/j.envint.2021.106420. 33556912

[R82] Marsland AL, Gianaros PJ, Kuan DCH, Sheu LK, Krajina K, Manuck SB, 2015. Brain morphology links systemic inflammation to cognitive function in midlife adults. Brain Behav. Immun. 48, 195–204. 10.1016/j.bbi.2015.03.015. 25882911 PMC4508197

[R83] Martin M, 2011. Cutadapt removes adapter sequences from high-throughput sequencing reads [next generation sequencing. small RNA; microRNA; adapter removal] 17 (1), 3. 10.14806/ej.17.1.200, 2011.

[R84] Martinowich K, Lu B, 2008. Interaction between BDNF and serotonin: role in mood disorders. Neuropsychopharmacology 33 (1), 73–83. 10.1038/sj.npp.1301571. 17882234

[R85] Matthews K, Cavagnaro T, Weinstein P, Stanhope J, 2024. Health by design; optimising our urban environmental microbiomes for human health. Environ. Res. 257, 119226. 10.1016/j.envres.2024.119226. 38797467

[R86] McMurdie PJ, Holmes S, 2013. Phyloseq: an R package for reproducible interactive analysis and graphics of microbiome census data. PLoS One 8 (4), e61217. 10.1371/journal.pone.0061217. 23630581 PMC3632530

[R87] Misera A, Marlicz W, Podkówka A, Łoniewski I, Skonieczna-Żydecka K, 2024. Possible application of Akkermansia muciniphila in stress management. Microbiome Res Rep 3 (4), 48. 10.20517/mrr.2023.81. 39741949 PMC11684984

[R88] Mosquera FEC, Lizcano Martinez S, Liscano Y, 2024. Effectiveness of psychobiotics in the treatment of psychiatric and cognitive disorders: a systematic review of randomized clinical trials. Nutrients 16 (9), 1352. 10.3390/nu16091352. 38732599 PMC11085935

[R89] National Centers for Environmental Information, 2024. U.S. Local Climatological Data. https://www.ncei.noaa.gov.

[R90] National Oceanic and Atmospheric Administration, 2024. NOAA online weather data. https://www.weather.gov/wrh/Climate?wfo=sew.

[R91] Oksanen J, Simpson G, Blanchet FG, Kindt R, Legendre P, Minchin PR, O’Hara RB, Solymos P, Stevens MHH, Szoecs E, Wagner H, Barbour M, Bedward M, Bolker B, Borcard D, Carvalho G, Chirico M, De Caceres M, Durand S, Borman T, 2025. Vegan: community ecology package. In: Version R Package Version 2.6–10) CRAN. https://CRAN.R-project.org/package=vegan.

[R92] Ou Z, Deng L, Lu Z, Wu F, Liu W, Huang D, Peng Y, 2020. Protective effects of Akkermansia muciniphila on cognitive deficits and amyloid pathology in a mouse model of Alzheimer’s disease. Nutr. Diabetes 10 (1), 12. 10.1038/s41387-020-0115-8. 32321934 PMC7176648

[R93] Palmer M, Kuegler O, Christensen G, 2019. Washington’s forest resources, 2007–2016: 10-year Forest Inventory and Analysis report. 10.2737/PNW-GTR-976.

[R94] Patterson E, Tan HTT, Groeger D, Andrews M, Buckley M, Murphy EF, Groeger JA, 2024. Bifidobacterium longum 1714 improves sleep quality and aspects of well-being in healthy adults: a randomized, double-blind, placebo-controlled clinical trial. Sci. Rep. 14 (1), 3725. 10.1038/s41598-024-53810-w. 38355674 PMC10866977

[R95] Pearson AL, Pechal J, Lin Z, Benbow ME, Schmidt C, Mavoa S, 2020. Associations detected between measures of neighborhood environmental conditions and human microbiome diversity. Sci. Total Environ. 745, 141029. 10.1016/j.scitotenv.2020.141029. 32721621

[R96] Pearson AL, Rzotkiewicz A, Pechal JL, Schmidt CJ, Jordan HR, Zwickle A, Benbow ME, 2019. Initial evidence of the relationships between the human postmortem microbiome and neighborhood blight and greening efforts. Ann. Assoc. Am. Geogr. 109 (3), 958–978. 10.1080/24694452.2018.1519407.

[R97] Prescott SL, Logan AC, 2017. The Secret Life of your Microbiome: Why Nature and Biodiversity are Essential to Health and Happiness. New Society Publishers.

[R98] Quast C, Pruesse E, Yilmaz P, Gerken J, Schweer T, Yarza P, Peplies J, Glöckner FO, 2013. The SILVA ribosomal RNA gene database project: improved data processing and web-based tools. Nucleic Acids Res. 41 (D1), D590–D596. 10.1093/nar/gks1219. 23193283 PMC3531112

[R99] R Core Team, 2024. R: a Language and Environment for Statistical Computing. R Foundation for Statistical Computing. https://www.R-project.org/.

[R100] Roberts DW, 2023. Labdsv: ordination and multivariate analysis for ecology. In: Version R Package Version 2.1–0) CRAN. https://CRAN.R-project.org/package=labdsv.

[R101] Rothschild D, Weissbrod O, Barkan E, Kurilshikov A, Korem T, Zeevi D, Costea PI, Godneva A, Kalka IN, Bar N, Shilo S, Lador D, Vila AV, Zmora N, Pevsner-Fischer M, Israeli D, Kosower N, Malka G, Wolf BC, Segal E, 2018. Environment dominates over host genetics in shaping human gut microbiota. Nature 555 (7695), 210. 10.1038/nature25973. 29489753

[R102] Ryan RP, Monchy S, Cardinale M, Taghavi S, Crossman L, Avison MB, Berg G, van der Lelie D, Dow JM, 2009. The versatility and adaptation of bacteria from the genus Stenotrophomonas. Nat. Rev. Microbiol. 7 (7), 514–525. 10.1038/nrmicro2163. 19528958

[R103] Sarkar A, Harty S, Lehto SM, Moeller AH, Dinan TG, Dunbar RIM, Cryan JF, Burnet PWJ, 2018. The microbiome in psychology and cognitive neuroscience.Trends Cognit. Sci. 22 (7), 611–636. 10.1016/j.tics.2018.04.006. 29907531

[R104] Sarkar A, Lehto SM, Harty S, Dinan TG, Cryan JF, Burnet PWJ, 2016. Psychobiotics and the manipulation of bacteria-gut-brain signals. Trends Neurosci. 39 (11), 763–781. 10.1016/j.tins.2016.09.002. 27793434 PMC5102282

[R105] Savignac HM, Kiely B, Dinan TG, Cryan JF, 2014. Bifidobacteria exert strain-specific effects on stress-related behavior and physiology in BALB/c mice. Neuro Gastroenterol. Motil. 26 (11), 1615–1627. 10.1111/nmo.12427. 25251188

[R106] Savignac HM, Tramullas M, Kiely B, Dinan TG, Cryan JF, 2015. Bifidobacteria modulate cognitive processes in an anxious mouse strain. Behav. Brain Res. 287, 59–72. 10.1016/j.bbr.2015.02.044. 25794930

[R107] Schiavi E, Gleinser M, Molloy E, Groeger D, Frei R, Ferstl R, Rodriguez-Perez N, Ziegler M, Grant R, Moriarty Thomas F, Plattner S, Healy S, O’Connell Motherway M, Akdis Cezmi A, Roper J, Altmann F, van Sinderen D, O’Mahony L, 2016. The surface-associated exopolysaccharide of *Bifidobacterium longum* 35624 plays an essential role in dampening host proinflammatory responses and repressing local TH17 responses. Appl. Environ. Microbiol. 82 (24), 7185–7196. 10.1128/AEM.02238-16. 27736791 PMC5118929

[R108] Selway CA, Mills JG, Weinstein P, Skelly C, Yadav S, Lowe A, Breed MF, Weyrich LS, 2020. Transfer of environmental microbes to the skin and respiratory tract of humans after urban green space exposure. Environ. Int. 145, 106084. 10.1016/j.envint.2020.106084. 32977191

[R109] Sharma M, Wasan A, Sharma RK, 2021. Recent developments in probiotics: an emphasis on Bifidobacterium. Food Biosci. 41, 100993. 10.1016/j.fbio.2021.100993.

[R110] Sharma R, Gupta D, Mehrotra R, Mago P, 2021. Psychobiotics: the next-generation probiotics for the brain. Curr. Microbiol. 78 (2), 449–463. 10.1007/s00284-020-02289-5. 33394083

[R111] Si J, Hyena K, Ju YH, Ko G, 2022. Revisiting the role of Akkermansia muciniphila as a therapeutic bacterium. Gut Microbes 14 (1), 2078619. 10.1080/19490976.2022.2078619. 35613313 PMC9135416

[R112] Silva A.C. R.d., Lopes PM, Azevedo M.M. B.d., Costa DCM, Alviano CS, Alviano DS, 2012. Biological activities of a-pinene and β-pinene enantiomers. Molecules 17 (6), 6305–6316. https://www.mdpi.com/1420-3049/17/6/6305. 22634841 10.3390/molecules17066305 PMC6268778

[R113] Smith BJ, Miller RA, Ericsson AC, Harrison DC, Strong R, Schmidt TM, 2019. Changes in the gut microbiome and fermentation products concurrent with enhanced longevity in acarbose-treated mice. BMC Microbiol. 19 (1), 130. 10.1186/s12866-019-1494-7. 31195972 PMC6567620

[R114] Sobko T, Liang S, Cheng WHG, Tun HM, 2020. Impact of outdoor nature-related activities on gut microbiota, fecal serotonin, and perceived stress in preschool children: the play&grow randomized controlled trial. Sci. Rep. 10 (1), 21993. 10.1038/s41598-020-78642-2. 33319792 PMC7738543

[R115] Song H, Zou J, Sun Z, Pu Y, Qi W, Sun L, Li Q, Yuan C, Wang X, Gao X, Zheng Y, 2025. Nasal microbiome in relation to olfactory dysfunction and cognitive decline in older adults. Transl. Psychiatry 15 (1), 122. 10.1038/s41398-025-03346-y. 40185726 PMC11971419

[R116] Sudo N, Chida Y, Aiba Y, Sonoda J, Oyama N, Yu X-N, Kubo C, Koga Y, 2004. Postnatal microbial colonization programs the hypothalamic–pituitary–adrenal system for stress response in mice. J. Physiol. 558 (1), 263–275. 10.1113/jphysiol.2004.063388. 15133062 PMC1664925

[R117] Sun J, Zhang Y, Kong Y, Ye T, Yu Q, Kumaran Satyanarayanan S, Su K-P, Liu J, 2022. Microbiota-derived metabolite Indoles induced aryl hydrocarbon receptor activation and inhibited neuroinflammation in APP/PS1 mice. Brain Behav. Immun. 106, 76–88. 10.1016/j.bbi.2022.08.003. 35961580

[R118] Sun Y, Meng Y, Ou Z, Li Y, Zhang M, Chen Y, Zhang Z, Chen X, Mu P, Norbäck D, Zhao Z, Zhang X, Fu X, 2022. Indoor microbiome, air pollutants and asthma, rhinitis and eczema in preschool children – a repeated cross-sectional study. Environ. Int. 161, 107137. 10.1016/j.envint.2022.107137. 35168186

[R119] Taufer F, Pálsdóttir AM, Hedblom M, 2025. Psychological and physiological responses to smells from nature—potential health benefits for urban dwellers. npj Urban Sustainability 5 (1), 80. 10.1038/s42949-025-00274-0.

[R120] Tennant R, Hiller L, Fishwick R, Platt S, Joseph S, Weich S, Parkinson J, Secker J, Stewart-Brown S, 2007. The Warwick-Edinburgh Mental Well-being Scale (WEMWBS): development and UK validation. Health Qual. Life Outcome 5 (1), 63. 10.1186/1477-7525-5-63. PMC2222612 18042300

[R121] Thangaleela S, Sivamaruthi BS, Kesika P, Bharathi M, Chaiyasut C, 2022. Nasal microbiota, olfactory health, neurological disorders and aging—A review. Microorganisms 10 (7). 10.3390/microorganisms10071405. PMC9320618 35889124

[R122] Tian P, Bastiaanssen TFS, Song L, Jiang B, Zhang X, Zhao J, Zhang H, Chen W, Cryan JF, Wang G, 2021. Unraveling the microbial mechanisms underlying the psychobiotic potential of a Bifidobacterium breve strain. Mol. Nutr. Food Res. 65 (8), 2000704. 10.1002/mnfr.202000704. 33594816

[R123] Trapnell PD, Campbell JD, 1999. Private self-consciousness and the five-factor model of personality: distinguishing rumination from reflection. J. Personality Soc. Psychol. 76 (2), 284–304. 10.1037/0022-3514.76.2.284. 10074710

[R124] Ulrich RS, Simons RF, Losito BD, Fiorito E, Miles MA, Zelson M, 1991. Stress recovery during exposure to natural and urban environments. J. Environ. Psychol. 11 (3), 201–230. 10.1016/S0272-4944(05)80184-7.

[R125] United Nations, D. o. E. S. A., Population Division, 2019. World Urbanization Prospects 2018: Highlights (ST/ESA/SER.A/421).

[R141] van den Berg AE, Joye Y, de Vries S. 2018. Health benefits of nature. In: Environmental Psychology. John Wiley & Sons, Ltd, pp. 55–64. https://onlinelibrary.wiley.com/doi/abs/10.1002/9781119241072.ch6.

[R126] Varadarajan S, Herchet M, Mack M, Hofmann M, Bisle E, Sayer E, Kolassa I-T, 2025. Salutogenic effects of greenspace exposure: an integrated biopsychological perspective on stress regulation, mental and physical health in the urban population. Open Psychology 7 (1), 20240003.

[R127] Vásquez-Pérez JM, González-Guevara E, Gutiérrez-Buenabad D, Martínez-Gopar PE, Martinez-Lazcano JC, Cárdenas G, 2024. Is nasal dysbiosis a required component for neuroinflammation in major depressive disorder? Mol. Neurobiol. 10.1007/s12035-024-04375-2. 39120823

[R128] Volk JK, Nyström EEL, van der Post S, Abad BM, Schroeder BO, Johansson Å, Svensson F, Jäverfelt S, Johansson MEV, Hansson GC, Birchenough GMH, 2019. The Nlrp6 inflammasome is not required for baseline colonic inner mucus layer formation or function. J. Exp. Med. 216 (11), 2602–2618. 10.1084/jem.20190679. 31420376 PMC6829596

[R129] Wall R, Cryan JF, Ross RP, Fitzgerald GF, Dinan TG, Stanton C, 2014. Bacterial neuroactive compounds produced by psychobiotics. In: Lyte M, Cryan JF (Eds.), Microbial Endocrinology: the microbiota-gut-brain Axis in Health and Disease. Springer, New York, pp. 221–239. 10.1007/978-1-4939-0897-4_10. 24997036

[R130] Watson D, Clark LA, Tellegen A, 1988. Development and validation of brief measures of positive and negative affect: the PANAS scales. J. Personality Soc. Psychol. 54 (6), 1063–1070. 10.1037/0022-3514.54.6.1063. 3397865

[R131] Watson S, Mackin P, 2006. HPA axis function in mood disorders. Psychiatry 5 (5), 166–170.

[R132] White MP, Elliott LR, Grellier J, Economou T, Bell S, Bratman GN, Cirach M, Gascon M, Lima ML, Lõhmus M, 2021. Associations between green/blue spaces and mental health across 18 countries. Sci. Rep. 11 (1), 8903. 33903601 10.1038/s41598-021-87675-0 PMC8076244

[R133] Wickham H, 2016. ggplot2: Elegant Graphics for Data Analysis. Springer-Verlag. https://ggplot2.tidyverse.org.

[R134] Yaghoubfar R, Behrouzi A, Ashrafian F, Shahryari A, Moradi HR, Choopani S, Hadifar S, Vaziri F, Nojoumi SA, Fateh A, Khatami S, Siadat SD, 2020. Modulation of serotonin signaling/metabolism by Akkermansia muciniphila and its extracellular vesicles through the gut-brain axis in mice. Sci. Rep. 10 (1), 22119. 10.1038/s41598-020-79171-8. 33335202 PMC7747642

[R135] Zhang M, Tang H, Yuan Y, Ou Z, Chen Z, Xu Y, Fu X, Zhao Z, Sun Y, 2023. The role of indoor microbiome and metabolites in shaping children’s nasal and oral microbiota: a pilot multi-omic analysis. Metabolites 13 (10), 1040. https://www.mdpi.com/2218-1989/13/10/1040. 37887365 10.3390/metabo13101040 PMC10608577

[R136] Zhang Y-D, Zhou G-L, Wang L, Browning MHEM, Markevych I, Heinrich J, Knibbs LD, Zhao T, Ding Y, Chen S, Liu K-K, Dadvand P, Dong G-H, Yang B-Y, 2024. Greenspace and human microbiota: a systematic review. Environ. Int. 187, 108662. 10.1016/j.envint.2024.108662. 38653130

[R137] Zheng X, Dai X, Zhu Y, Yang J, Jiang H, Dong H, Huang L, 2022. (Meta)Genomic analysis reveals diverse energy conservation strategies employed by globally distributed Gemmatimonadota. mSystems 7 (4). 10.1128/msystems.00228-22 e00228–00222. 35913193 PMC9426454

[R138] Zhou Y, Chen Y, He H, Peng M, Zeng M, Sun H, 2023. The role of the indoles in microbiota-gut-brain axis and potential therapeutic targets: a focus on human neurological and neuropsychiatric diseases. Neuropharmacology 239, 109690. 10.1016/j.neuropharm.2023.109690. 37619773

[R139] Zhu Y, Chen B, Zhang X, Akbar MT, Wu T, Zhang Y, Zhi L, Shen Q, 2024. Exploration of the Muribaculaceae family in the gut microbiota: diversity, metabolism, and function. Nutrients 16 (16). 10.3390/nu16162660. PMC11356848 39203797

[R140] Zuccarella-Hackl C, Princip M, Auschra B, Meister-Langraf RE, Barth J, von Känel R, 2023. Association of positive psychological well-being with circulating inflammatory markers: a systematic review and meta-analysis. Neurosci. Biobehav. Rev. 150, 105186. 10.1016/j.neubiorev.2023.105186. 37076058

