# Supplementary material for: Associations of forest vs. urban environmental exposure with well-being and nasal microbiome composition: An exploratory pilot study
Source: Environ Res. Author manuscript; Available in PMC 2026 Mar 1. (PMC12950270; doi:10.1016/j.envres.2025.123582)
Supplement: MMC1 [file NIHMS2134036-supplement-MMC1.docx]

**Supplementary Material**

**
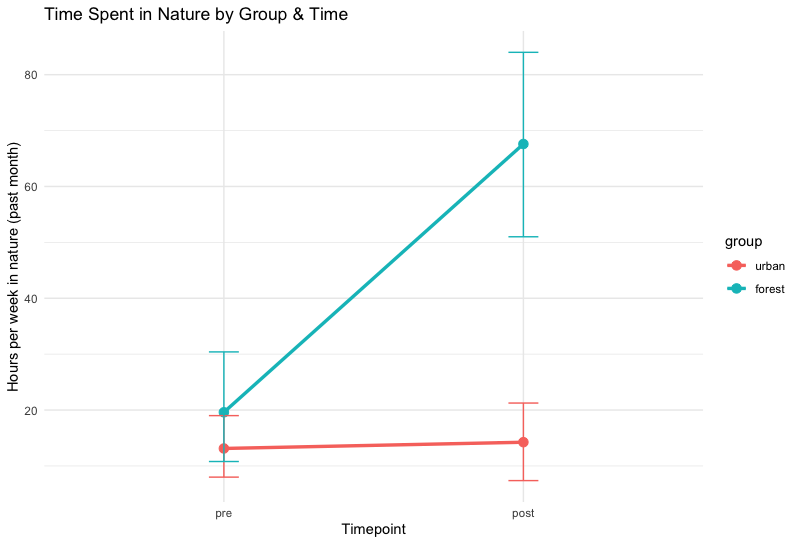
**

**Fig. S1. Self-Reported Hours Per Week Spent in Nature During the Month Preceding Assessment Stratified by Group and Time.** The forest group reported an increase in nature exposure at the post-exposure vs. pre-exposure timepoint, whereas the urban group showed minimal change over time. Points represent group means, lines connect means across time within groups, and error bars indicate 95% confidence intervals.

**
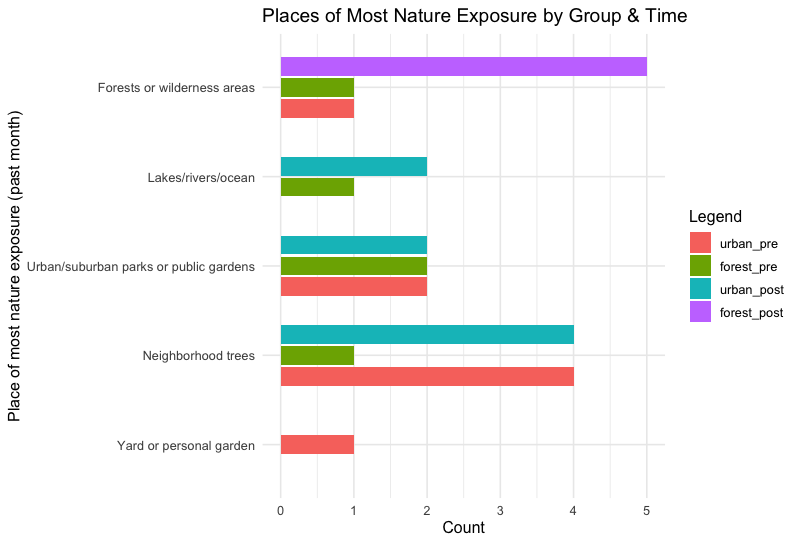
**

**Fig. S2. Self-Reported Locations of Most Frequent Nature Exposure During the Month Preceding Assessment Stratified by Group and Time.** Participants in the forest group unanimously reported forests or wilderness areas as their primary setting for nature exposure post-exposure, whereas the urban group reported a mix of locations. Bar lengths represent the number of participants who indicated each environment type.

**Table S1**

| **Measure (Survey)** | **Mean Score (SD)** | | | |
| --- | --- | --- | --- | --- |
|  | **Urban, Pre** | **Urban, Post** | **Forest, Pre** | **Forest, Post** |
| **Positive Affect (PANAS)** | 28.88 (5.36) | 28.75 (6.14) | 33.40 (3.85) | 41.20 (6.76) |
| **Negative Affect (PANAS)** | 17.25 (4.43) | 16.88 (2.36) | 24.40 (8.56) | 15.80 (5.40) |
| **Rumination (RRQ)** | 3.05 (0.81) | 2.85 (0.67) | 3.47 (0.36) | 2.60 (0.58) |
| **Well-being (WEMWBS)** | 46.50 (7.45) | 46.75 (11.42) | 48.40 (7.99) | 56.80 (4.60) |

**Summary of Mean Self-report Survey Scores by Group and Timepoint.**

*Note.* SD = standard deviation.

**Table S2**

| **Measure (Survey)** | **Group** | | | | **Time** | | | | **Group x Time** | | | |
| --- | --- | --- | --- | --- | --- | --- | --- | --- | --- | --- | --- | --- |
|  |  | **95% CI** | |  |  | **95% CI** | |  |  | **95% CI** | |  |
|  | ***β*** | **Lower** | **Upper** | ***p*** | ***β*** | **Lower** | **Upper** | ***p*** | ***β*** | **Lower** | **Upper** | ***p*** |
| **Positive Affect (PANAS)** | 4.53 | -1.80 | 10.86 | .18 | -0.13 | -4.23 | 3.98 | .95 | 7.93 | 1.31 | 14.54 | .04 |
| **Negative Affect (PANAS)** | 7.15 | 1.38 | 12.92 | .03 | -0.38 | -3.66 | 2.91 | .83 | -8.23 | -13.52 | -2.93 | .01 |
| **Rumination (RRQ)** | 0.41 | -0.33 | 1.16 | .29 | -0.20 | -0.49 | 0.09 | .20 | -0.67 | -1.13 | -0.20 | .02 |
| **Well-being (WEMWBS)** | 1.90 | -7.75 | 11.55 | .70 | 0.25 | -5.04 | 5.54 | .93 | 8.15 | -0.38 | 16.68 | .09 |

**Linear Mixed Effects Model Results for Change in Self-report Well-being Outcomes by Group, Time, and Group x Time Interaction.** Significant group x time interactions indicate that change over time in positive affect (PANAS), negative affect (PANAS), and rumination (RRQ) differed between groups, while the between-group difference in well-being (WEMWBS) change was only trend-level..

*Note. β =* expected mean difference in survey score. CI = confidence interval.

**Fig. S3. Alpha Rarefaction Curves Based on Observed Bacterial ASVs.** Curves approaching a plateau suggest adequate sampling depth for capturing bacterial richness of samples. Each gray line represents one sample. Short vertical segments along each line reflect variation across multiple rarefaction iterations at the same sequencing depth for a given sample. The bold black line represents the median.

**Table S3**

| **⍺-Diversity Measure** | **Mean (SD)** | | | |
| --- | --- | --- | --- | --- |
|  | **Urban, Pre** | **Urban, Post** | **Forest, Pre** | **Forest, Post** |
| **Richness (ASVs)** | 164.13 (71.93) | 170.75 (19.15) | 221.20 (57.56) | 322.60 (159.11) |
| **Shannon Diversity Index** | 2.26 (0.47) | 2.15 (0.43) | 2.44 (0.71) | 2.33 (0.78) |

**Summary of Nasal Bacterial ⍺-diversity Measures by Group and Timepoint.**

*Note.* SD = standard deviation.

**Table S4**

| **⍺-Diversity Measure** | **Group** | | | | | **Time** | | | | **Group x Time** | | | |
| --- | --- | --- | --- | --- | --- | --- | --- | --- | --- | --- | --- | --- | --- |
|  |  | **95% CI** | |  |  | | **95% CI** | |  |  | **95% CI** | |  |
|  | ***β*** | **Lower** | **Upper** | ***p*** | ***β*** | | **Lower** | **Upper** | ***p*** | ***β*** | **Lower** | **Upper** | ***p*** |
| **Richness (ASVs)** | 57.08 | -36.19 | 150.34 | .24 | 6.63 | | -67.78 | 81.03 | .87 | 94.78 | -25.20 | 214.75 | .15 |
| **Shannon Diversity Index** | 0.18 | -0.46 | 0.83 | .59 | -0.11 | | -0.29 | 0.07 | .25 | -0.01 | -0.29 | 0.28 | .97 |

**Linear Mixed Effects Model Results for Change in Bacterial ⍺-diversity Measures by Group, Time, and Group x Time Interaction.** Lack of significant interaction terms indicates that change over time in richness and Shannon diversity index did not differ across groups.

*Note. β =* expected mean difference in ɑ-diversity measure. CI = confidence interval.

**Table S5**

|  |  |  | **95% CI** | |  |
| --- | --- | --- | --- | --- | --- |
| **ɑ-Diversity Measure** | **Predictor** | ***β*** | **Lower** | **Upper** | ***p*-value** |
| **Richness (ASVs)** | Group (forest) | 136.58 | -2.92 | 276.08 | .05 |
|  | Baseline richness | 0.27 | -0.74 | 1.27 | .57 |
| **Shannon Diversity Index** | Group (forest) | 0.01 | -0.33 | 0.35 | .95 |
|  | Baseline Shannon | 0.92 | 0.61 | 1.23 | <.01 |

**ANCOVA Results Examining Post-Exposure Bacterial ɑ-Diversity as a Function of Exposure Group, Adjusting for Baseline ɑ-Diversity.** After adjusting for baseline values, the forest group showed a trend toward higher post-exposure richness than the urban group but no difference in Shannon diversity.

*Note. β =* regression coefficient representing the estimated mean group difference in ɑ-diversity measure post-exposure after adjusting for baseline, or the expected mean change in post-exposure ɑ-diversity based on baseline value alone across all participants; CI = confidence interval.

**Fig. S4. NMDS Ordinations of Nasal Bacterial Community Composition Based on Bray-Curtis Dissimilarity.** Panels show NMDS ordinations based on Bray-Curtis dissimilarity, a weighted measure that incorporates both the presence and relative abundance of taxa. Panels A and B represent comparisons of overall bacterial community composition across timepoints (within groups). Panels C and D represent comparisons of overall bacterial community composition across groups (within timepoints). Each point represents a single sample. Ellipses indicate 95% confidence intervals around group centroids. No statistically significant differences in overall nasal bacterial community composition were detected across any comparisons.

*Note. p-*values provided with each panel are based on PERMANOVA.

**Table S6**

**Significant Indicator Taxa of the Forest Group Post-Exposure.** Taxonomic assignments are provided to the most specific level of classification available in our analyses. Mean relative abundances reflect the average proportion of total sequences assigned to each taxon across all forest group post-exposure samples. All identified taxa were present at < 1% relative abundance.

**Fig. S5. Differentially abundant bacterial taxa in the urban group from pre- to post-exposure.** Log_2_ fold changes in ASV relative abundance over the study period for 20 enriched and 11 depleted taxa (FDR-adjusted *p* < .05). Positive values (blue bars) indicate taxa that increased in relative abundance in the urban group’s nasal microbiomes from pre- to post-exposure, while negative values (gray bars) indicate taxa that decreased in relative abundance.

**Table S7**

|  |  | **Robust Wald 95% CI** | |  |
| --- | --- | --- | --- | --- |
| **Measure (Survey)** | ***β*** | **Lower** | **Upper** | ***p*-value** |
| **Positive Affect (PANAS)** | 0.03 | 0.008 | 0.04 | .01 |
| **Negative Affect (PANAS)** | -0.03 | -0.06 | -0.004 | .03 |
| **Rumination (RRQ)** | -0.0005 | -0.002 | 0.001 | .50 |
| **Well-being (WEMWBS)** | 0.02 | -0.007 | 0.05 | .14 |

**Associations of Change in Self-report Survey Score with Increased Nasal Bacterial Richness.** Results from simple linear regression models describing the relationship between change in self-report affect, rumination, and well-being survey scores and increased nasal bacterial richness. Significant associations were detected linking affective (positive and negative) improvements with increased nasal bacterial richness.

*Note. β =* expected mean difference in survey score per additional unique ASV. CI = confidence interval.

**Fig. S6. Q-Q plot of standardized residuals for the regression of positive affect change on richness change.** Residuals exhibit heavy-tail deviations, indicating some values may be more extreme than expected under normality.

**Fig. S7. Q-Q plot of standardized residuals for the regression of negative affect change on richness change.** Residuals exhibit moderate heavy-tail deviations, indicating some values may be more extreme than expected under normality.

**Table S8**

|  |  | **Robust Wald 95% CI** | |  |
| --- | --- | --- | --- | --- |
| **Measure (Survey)** | ***β*** | **Lower** | **Upper** | ***p*-value** |
| **Positive Affect (PANAS)** | 8.43 | -5.16 | 22.01 | .20 |
| **Negative Affect (PANAS)** | -10.80 | -22.17 | 0.58 | .06 |
| **Rumination (RRQ)** | -0.29 | -1.80 | 1.23 | .68 |
| **Well-being (WEMWBS)** | 9.00 | 0.17 | 17.82 | .05 |

**Associations of Change in Self-report Survey Score with Increased Shannon Diversity Index (SDI).** Results from simple linear regression models describing the relationship between change in self-reported affect, rumination, and well-being survey scores and increased nasal bacterial SDI. Marginally significant associations were detected linking decreased negative affect and increased well-being with increased SDI.

*Note. β =* expected mean difference in survey score per unit increase in SDI. CI = confidence interval.
